# Measuring planetary health knowledge: Development and psychometric evaluation of a taxonomy-based test

**DOI:** 10.1371/journal.pone.0359156

**Published:** 2026-10-06

**Authors:** Franziska Rees, Oliver Wilhelm

**Affiliations:** Department of Individual Differences and Psychological Assessment, Ulm University, Ulm, Germany; Charite Universitatsmedizin Berlin, GERMANY

## Abstract

Planetary Health Knowledge (PHK) refers to knowledge about global conditions and interrelations between the health of the planet, human health, and human civilization. In times of various humanitarian and environmental crises, knowledge about and awareness of these problems become increasingly important. This is reflected in the Sustainable Development Goals and various Global Citizenship Education programs that emphasize the importance of knowledge about global challenges as a key characteristic in promoting sustainability. Despite its relevance, a comprehensive, psychometrically evaluated measure of PHK is currently lacking. Based on the recently proposed PHK Taxonomy, we developed a comprehensive PHK item pool of approximately 500 knowledge items targeting knowledge about global issues in seven domains: *Health*, *Nutrition*, *Environment*, *Safety*, *Education*, *Standard of Living*, and *Political & Economic systems*. The item pool was evaluated in seven domain-specific psychometric studies using a balanced-incomplete-block-design, administered to a total of *N* = 2204 participants. Items suitable for general population samples were selected based on psychometric characteristics. Construct validity was examined via associations with general knowledge (gc), global citizenship identification (GCI), sustainable behavior, educational attainment, subjective knowledge, information seeking frequency, and party affiliation. Across all domains, PHK showed moderate to strong associations with gc (*r* = .539 − .736) and educational attainment, and weaker associations with GCI (*r* = .238 − .416). Associations with sustainable behavior, subjective knowledge, and information seeking-frequency were small to negligible. The resulting domain-specific PHK item sets provide psychometrically evaluated PHK measures for assessing PHK in general population samples. Finally, we discuss implications for knowledge assessment and future applications in research and evaluation.

## 1. Introduction

Humanity faces numerous interconnected environmental and societal crises, such as climate change, poverty, pollution, food insecurity, and global health inequalities. These challenges are increasingly viewed as interconnected threats to both the environment and to human civilization [1]. Knowledge about such crises—specifically about their magnitude, distribution, causes, consequences, and interrelations—is an important prerequisite for identifying problems, evaluating possible responses, and making informed decisions [2–4]. We refer to this body of knowledge as *Planetary Health Knowledge* (PHK). PHK addresses the current state and the health of the environment, human individuals, and human societies, as well as their interactions [5], and may contribute to the transition of the planet and human civilization to a more sustainable future [2,5].

Importantly, knowledge alone is not a sufficient predictor of sustainable behavior or for responsible citizenship [6]. Rather, educational and political frameworks commonly highlight certain knowledge alongside skills, values, and competencies as important characteristics to act as a global citizen, i.e., that enable individuals to engage in actions supporting environmental and social sustainability [2,3,7–9]. The relevance of these characteristics is reflected in various frameworks such as the Sustainable Development Goals (SDGs), Planetary Health [10], and Global Citizenship Education [8,9] which call for people to acquire relevant knowledge and skills to promote sustainable development [11]. However, none of them provides an explicit specification and definition of the knowledge that constitutes PHK [5]. Although these frameworks differ in the use of terminology, disciplinary origins, perspectives, and intended applications, they address a wide range of environmental, social, health-related, political, and economic challenges, that are deemed the most important issues that humanity currently faces [5].

Fostering PHK raises two important questions: First, how can the scope of relevant knowledge be defined, and second, how can such knowledge be assessed reliably and validly? To address the first question, previous research has integrated content from major frameworks such as the SDGs [11], Planetary Health Framework [10], and Global Citizenship Education frameworks [8] in order to specify the scope of relevant knowledge and developed the PHK taxonomy (Fig 1). This taxonomy organizes the content from all frameworks in seven content-specific but interrelated domains. Establishing such a taxonomy is an indispensable step in test development because it specifies the scope of the content of a domain that a test is intended to represent.

**Fig 1:**
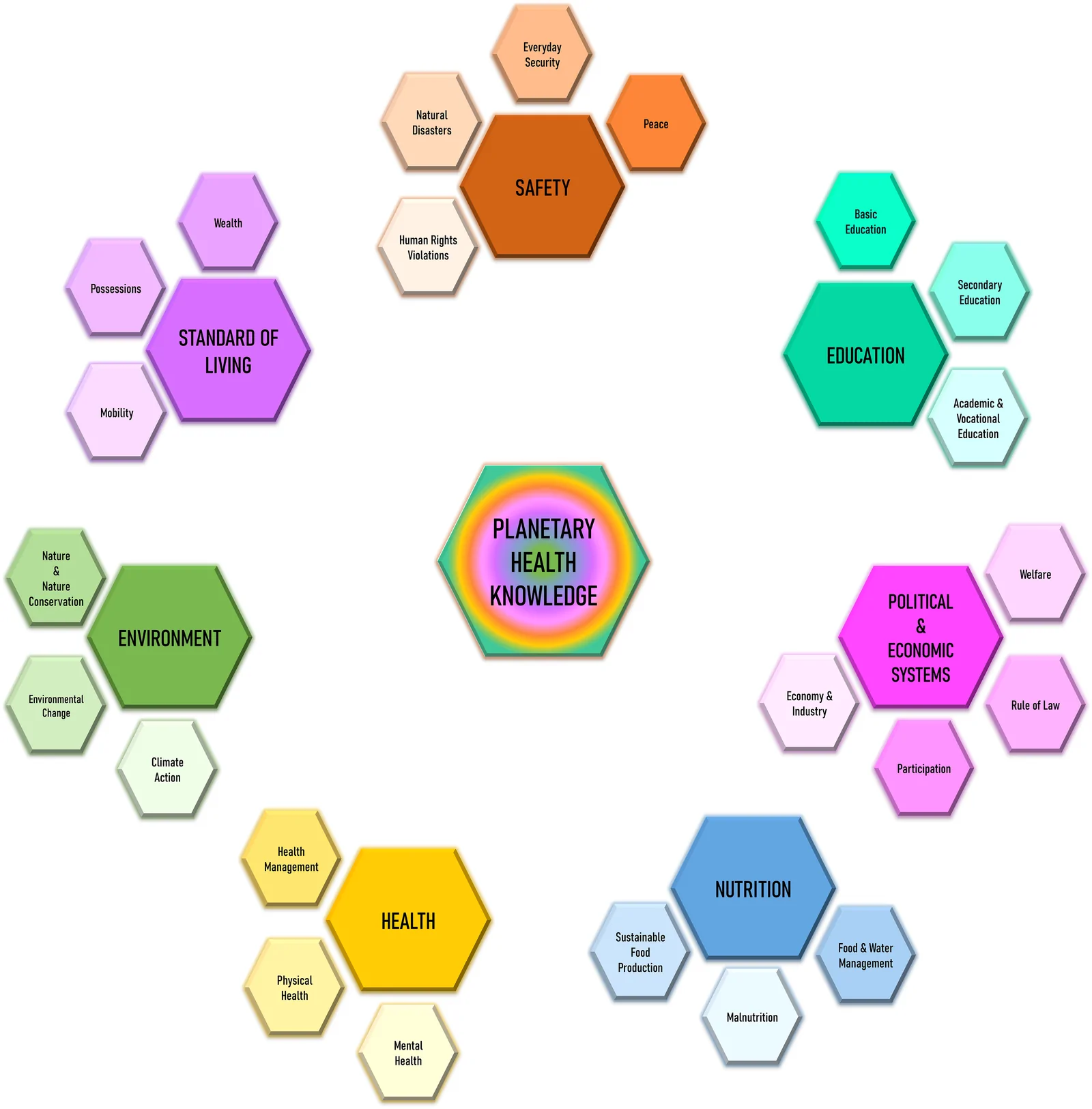
Planetary Health Knowledge Taxonomy. An illustration incorporating the facet structure can be found on the OSF.

Second, to systematically assess and investigate PHK requires psychometrically sound assessment tools. Such PHK tests serve educational and diagnostic purposes. By assessing individuals’ understanding about the interrelation of human health and environmental and social issues, these tests may be used to identify knowledge gaps and misconceptions regarding the intricate link between the human disruption of the Earth’s ecosystems and global health outcomes. They may further provide a basis for tailoring and evaluating educational curricula and public health campaigns. Furthermore, high-quality assessments cultivate critical thinking and self-awareness among students and professionals, fostering a sense of agency that transforms theoretical knowledge into pro-environmental behaviors. Ultimately, validating and expanding planetary health literacy through standardized testing establishes a foundational metric necessary for monitoring community readiness and empowering individuals to advocate for sustainable, evidence-based policy solutions. Acknowledging the importance of knowledge about planetary health highlights the demand for psychometrically sound knowledge tests to assess individuals’ PHK. As no comprehensive PHK tests currently exist, this study closes this gap by developing and evaluating a comprehensive PHK item pool.

Before turning to test development, we first, provide a brief overview of declarative knowledge, second introduce PHK and the PHK taxonomy conceptually situate PHK within the framework of declarative knowledge, and review existing knowledge tests in the area of PHK. As no instrument captures the complete scope of PHK, we develop and psychometrically evaluate a comprehensive PHK item pool in the present contribution.

### 1.1. Declarative knowledge

Recent research has identified declarative knowledge as a key indicator of crystallized intelligence (gc) [12]. We will use the term *declarative knowledge* to refer to the body of knowledge accumulated across the lifetime through both, formal (i.e., structured and institution-based learning experiences) and informal (i.e., unstructured, experience-based learning in everyday life) learning [13,14]. As such, a key characteristic of knowledge is the existence of objectively veridical correct responses. Traditional gc-tests predominantly assess knowledge acquired through formal education and content is often derived from educational curricula. Knowledge about current salient events and knowledge acquired through everyday experiences is often underrepresented in such tests and thus, exempt from larger multivariate studies.

With respect to this broadly conceptualized declarative knowledge, structural investigations revealed four broad knowledge factors in the domains of humanities, life sciences, natural sciences, and social sciences. Below such higher-order factors, 34 more subject-specific domains such as biology, architecture, and geography can be subsumed [15].

### 1.2. Planetary health knowledge

PHK is conceptualized as a domain-specific form of declarative knowledge. Specifically, PHK refers to factual and conceptual knowledge about the current state of the world, current global issues, and the causes and consequences of human and societal health in relation to planetary systems [5]. PHK is not based on traditional curricular knowledge domains. As specific excerpt of general knowledge that integrates various domains of declarative knowledge such as biology, ecology, economics, geography, law, medicine, psychology, and politics, PHK spans across traditional knowledge domains. As PHK addresses current global issues, it includes knowledge about current global events contemporary global developments that can be acquired not only through formal learning but also through informal learning experiences and media exposure. The scope of knowledge relevant for PHK was recently defined by the development of the PHK taxonomy [5]. The seven PHK domains emerged from a literature-based integration and classification of the content derived from established frameworks [5]. As the SDGs provide the broadest structured collection of environmental, social, political, and economic challenges altogether, they served as initial guiding framework. At first, content elements were extracted from the goals and targets, while separating multi-aspect elements and eliminating redundant elements. The resulting content elements were then, grouped into a hierarchical structure of thematic facets, which were grouped into broader subdomains and domains. Thereby, a comprehensive coverage, thematic distinctiveness, parsimony, usefulness, and replicability was attempted. The preliminary structure was then, supplemented with content from other frameworks such as the Planetary Health framework and global citizenship frameworks. Last, two independent raters assigned the content elements again to the emerging categories, while disagreements on the ratings were used to refine the definitions and repeat the assignments. Evidently, the reduction of strong conceptual and empirical collinearity between neighboring concepts such as the SDGs, PHK, and the like warrants careful scrutiny.

The PHK taxonomy [5], grounded in concepts and frameworks such as the *Sustainable Development Goals*, *Planetary Health*, and *Global Citizenship*, outlines seven interrelated domains: i. *Health* (covering physical and mental health as well as global health management), ii. *Nutrition* (addressing global food security, sustainable food systems, and malnutrition), iii. *Environment* (encompassing the condition of natural ecosystems, climate change, and mitigation strategies), iv. *Safety* (focusing on global safety issues including wars, terrorism, violence, human rights violations, and threats of natural disasters), v. *Education* (examining access to education, quality of education, and its role in development), vi. *Standard of Living* (focusing on the standard of living of individuals by assessing global disparities in wealth, mobility, and well-being), and vii. *Political and Economic Systems* (analyzing governance structures, rule of law, economic conditions, and civic participation worldwide) (Fig 1). These domains represent global issues that manifest at a local, regional, national, or global scale. Considering these levels of manifestations in test design can allow for nuanced assessment of discrete topics and their interdependencies in varying degrees of granularity. However, integrating the different levels of granularity immensely increases the amount of information that needs consideration in compiling a knowledge test.

#### 1.2.1. Measures in the context of PHK.

In prior work a number of tests have been developed that cover parts of PHK but overarching tests considering all PHK domains are still pending. The *Gapminder Test*, for instance, covers all 17 SDGs using numerical stimuli. The test is mainly designed to highlight common misconceptions about development policies [16] and cannot be easily used as a standard achievement test. Another comprehensive instrument assesses knowledge about global characteristics and issues with 114 items across 13 domains (Environment, Health, Food, Energy, Arts & Culture, Religion, Distribution of Natural Characteristics, Relations among States, War & Armament, International Monetary and Trade Arrangements, Human Rights, Racial & Ethnic Issues, Population) [17]. Developed in the United States in the early 1980s, the test reflects a strong American-centric perspective and addresses global issues that are now outdated. Moreover, in contrast to our understanding of PHK, it also includes historical knowledge as well as domains such as religion, art, and culture—areas that might be relevant for human civilization but which do not belong into the field of PH.

The 30-item *sustainability knowledge test* [18] focuses on environmental, economic, and societal sustainability, leaving out human health and social systems issues. Similarly, the 25-item *world knowledge test* assesses knowledge across six domains emphasizing soft skill knowledge over factual knowledge [19].

Several available instruments that cover parts of PHK are tailored to measure environmental knowledge and they cover subdomains such as climate change, energy consumption, waste management, water, industrial pollution, health, and general public issues with lengths varying from 14 to 64 items [20–25]. Recently, environmental knowledge was found to be highly correlated with general knowledge [22]. More specific tests include the 48-items *International Ocean Literacy* test [26] focusing on the conceptual understanding concerning oceans in seven domains. Despite their breadth, these instruments focus on environmental aspects—in case of ocean literacy with a very specific scope—and knowledge related to human-induced climate change. Additionally, they focus predominantly on anthropocentric impacts on the environment, but they insufficiently address bidirectional interactions between environment and humans. Consequently, they do not capture, how (changing) environmental conditions affect human health, social structures, and living-conditions.

Health knowledge covers some aspects of PHK and there are a number of instruments to assess health knowledge across domains such as physical illnesses, mental health, and nutrition [27,28]. More specific instruments focus on topics such as antibiotics and the handling of antibiotic resistances [29]. Interestingly, no knowledge tests exist that cover knowledge about global cooperation, global economic issues, global variations in the standard of living, or human rights.

To the best of our knowledge, no instrument exists that comprehensively integrates factual knowledge about global issues across all PHK domains. Particularly, social dimensions are often underrepresented, highlighting the paucity of broad, integrative PHK measures. In contrast to broader competence-oriented frameworks and large-scale assessments [e.g., 9] that combine knowledge with problem solving reasoning, and evaluative skills, we focus on declarative knowledge in order to assess PHK as comparatively distinct cognitive ability and mostly independent from additional reasoning or evaluative processes.

### 1.3. Test development – Current study

In the present study, we develop and psychometrically evaluate a broad and comprehensive item pool for assessing PHK. Test development follows established psychometric principles of construct definition, domain specification, and psychometric evaluation [30,31]. The first hurdle is to define the scope of the knowledge of interest and systematically develop items that match this definition to address content validity. Here, we build on the established PHK taxonomy [5], that conceptualizes PHK as a multidimensional construct comprising seven broad PHK domains that are assumed to be situated below an overarching general PHK factor. The item development was guided by this taxonomy in which each domain is further differentiated into three or four more specific subdomains. Given PHK covers enduring global structures and current global events, its assessment must capture stable conceptual frameworks and systemic interrelations as well as current global events. Consequently, we distinguish four interrelated aspects of PHK: *Prevalence knowledge* (Prev): knowing the extent to which a problem affects a group of persons; ii) *Global distribution knowledge* (Glob): knowing where problems are particularly prevalent or critical; iii) *Declarative knowledge* (Fact): factual and conceptual knowledge of relevant concepts and issues; and iv) *Comprehension knowledge* (Comp): insight into causal and systemic interrelations among global issues. Although conceptually distinct, these four aspects are hypothesized to reflect a common underlying knowledge factor. To comprehensively cover the PHK taxonomy, separate item sets were developed for each of the seven domains.

Further analyses focus on the internal structure in terms of structural validity, according to which, together with psychometric analyses, forms the basis for item selection in order to condense the measurement within each item set and thereby facilitate the administration of the tests.

To further examine construct validity by investigating the nomological network of PHK, we selected external criteria based on recent findings. Specifically, convergent validity is assessed using constructs for which we expect positive associations with PHK. First, we examine the relations with *crystallized intelligence* (gc). Given the very strong correlations between environmental and general knowledge [22], we expect similarly strong correlations between PHK and gc. Likewise, *global citizenship identification* (GCI) issues are considered a core feature of global citizenship [3,19,32] and has been found to be associated with greater world knowledge, global understanding and awareness of global issues [19]. We thus expect a positive, albeit more moderate, relation between PHK and GCI (i.e., the feeling of belonging to a global rather than national community). Furthermore, previous research found modest positive associations between environmental and sustainability knowledge and pro-environmental and prosocial behavior [e.g., 20,22,33,34]. Thus, we expect small to moderate positive associations between PHK and pro-environmental behavior, prosocial behavior, and civic engagement.

Additionally, we include *subjective knowledge* (occasionally also labeled “perceived knowledge”) and information seeking frequency as constructs related to PHK. Subjective knowledge reflects an individual’s perceived knowledge of a topic and has frequently been used as knowledge indicator in psychological research. However, it is presumably not a reliable proxy for objective knowledge, as it correlates more strongly with various self-report dimensions than with knowledge measures [35]. We therefore predict at most weak correlations between PHK and subjective knowledge. Similarly, information frequency might intuitively enhance objective knowledge because repeated exposure to informational content through media consumption and active information seeking is assumed to facilitate knowledge acquisition. However, empirical evidence suggests weak or no correlations with objective knowledge [36–38], but instead suggests positive associations with subjective knowledge [37]. Accordingly, we expect at most weak correlations between PHK and subjective knowledge and little or no correlation between PHK and information frequency. In line with expected small correlations, we predict both variables to have only little effect in the prediction of PHK.

Further, declarative knowledge is assumed to be acquired and accumulated by formal education, but also by informal learning experiences and media exposure across the life span [39]. Educational attainment is also correlated with knowledge—and cognitive abilities per se. As we expect PHK to be partly acquired through educational attainment and formal learning, we expect educational attainment to positively predict PHK.

Last, political orientation and party affiliation shape individuals’ exposure to information and trust in scientific sources [40,41]. Additionally, formal education has been found a predictor of political affiliation with higher education being associated with more left political positions [42]. We thus, expect to find systematic differences in PHK across the political spectrum, with higher PHK scores among individuals with more left-leaning or green political orientations.

## 2. Methods

### 2.1. Participants and procedure

Data collection took place in two waves using a block design, in which participants responded to only one of the PHK domains. For samples 1, 2, and 3 (domains P1: Health, P2: Nutrition, and P3: Environment), we collected data in July 2023. Data collection for item sets 4, 5, 6, and 7 (domains P4: Safety, P5: Education, P6: Standard of Living, and P7: Political and Economic Systems) took place in January 2024. Participants were recruited via the German panel provider Bilendi [43] and all participants provided informed consent. All samples were representative to the German adult population concerning binary sex and age (18–74 years). Education was approximately uniformly distributed across three levels of education (lower secondary degree or vocational track *Hauptschule*, middle secondary degree or intermediate track *Realschule*, and higher secondary degree or academic track *Gymnasium*).

Sample size was determined in an a-priori power analysis using semPower [44]. In this analysis, alpha was set equal to beta which was set to .05. We were interested in the power of a CFA in which two latent variables correlated at approximately .25. One latent variable was specified to have 30 indicators, while the other had nine loadings. All loadings were assumed to have a magnitude of around .5. Based on this configuration, the required sample size was determined to be approximately *N* = 300, ensuring sufficient statistical power for detecting the specified relationships.

Participants were excluded from data analyses in case they failed one or more of two attention checks and in case of low response times (< 12 minutes for samples 1–3 and < 15 minutes in samples 4–7 for completing the knowledge items). The final sample sizes ranged from *N* = 303 (P1) to *N* = 326 (P4) (see Table 1 for sample characteristics of all 7 samples).

**Table 1:** Sample characteristics of studies 1 to 7.

	Study	1	2	3	4	5	6	7
	PHK Domain	P1	P2	P3	P4	P5	P6	P7
	N (total)	303	304	308	326	324	325	314
Gender	Male	150	147	148	157	163	169	162
	Female	153	157	160	167	161	155	152
	Other	0	0	0	2	0	1	0
Age	M	46.96	48.11	47.29	46.11	46.24	46.49	45.87
	SD	14.99	14.73	15.77	15.22	15.32	15.42	15.52
Education	Low	104	98	101	110	106	109	107
	Middle	108	94	102	105	109	106	97
	High	106	104	98	111	109	110	110
Party Affiliation Germany	AfD	64	64	73	68	55	69	54
	CDU/CSU	49	59	58	56	76	70	68
	The Left	15	15	18	20	14	13	11
	FDP	16	14	9	9	10	14	15
	Greens	52	35	32	35	25	31	30
	Other/ No vote	58	69	63	66	70	61	57
	SPD	49	48	55	45	46	42	40

P1 = Health, P2 = Nutrition, P3 = Environment, P4 = Safety, P5 = Education, P6 = Standard of Living, P7 = Political & Economic Systems. Low Education = lower secondary degree or vocational track, Middle Education = middle secondary degree or intermediate track, High Education = higher secondary degree or academic track. AfD = Alternative for Germany, CDU/CSU = Christian Democratic /Social Union, FDP = Free Democratic Party, SPD = Social Democratic Party of Germany, The Left = The Left, Greens = Alliance 90/The Greens.

After providing informed consent, participants responded to items assessing sociodemographic status, behavior, attitudes, general knowledge, and Planetary Health Knowledge (PHK) of the respective domain. For knowledge items, all responses were presented in a randomized order. In case of items targeting numbers, the numbers were presented in ascending order, ensuring that each response option was equally frequent within one domain. As responses to all items were mandatory and only complete cases were used for analyses, no missing data occurred at the item level. In samples 1–3 participants received immediate feedback about whether their response was correct and in case it was not, about the correct answer. This feedback was not provided in samples 4–7.

#### 2.1.1. Ethical approval and informed consent.

The study was conducted according to the ethical guidelines for online studies of the German Society for Online Research (DGOF) and in accordance with the Declaration of Helsinki. The study involved an anonymous, non-invasive survey assessing knowledge. No personal or sensitive data were collected. Ethical approval was not required as per local legislation. Participants were recruited via a German online panel and provided informed consent.

### 2.2. Material

#### 2.2.1. Sociodemographic questions.

Participants gave information about their age, gender, educational attainment, federal state, and mother tongue.

#### 2.2.2. General knowledge.

We used a 12-item short form of a test that comprises the 4 broad knowledge areas *natural sciences*, *social sciences*, *humanities*, and *life sciences* each of which included 3 more specific domains (e.g., music, politics, biology) to assess general knowledge (gc) [45]. Each of the 12 knowledge domains was measured by one item (e.g., “Who composed the magic flute?”) in a single-choice format with one correct and three erroneous answers each. Responses were scored dichotomously (0 = incorrect, 1 = correct). Gc was modeled as a g-factor model.

#### 2.2.3. Global citizenship identification.

Global Citizenship Identification (GCI) was assessed using eight items from the 20-item GCI scale [4]. A sample item reads “It is my responsibility to actively advocate for global issues”. The items should cover identification with *normative environment*, g*lobal awareness*, g*lobal citizenship*, *social justice*, and *environmental sustainability* with a 7-point Likert-type scale from 1 = *strongly disagree* to 7 = *strongly agree*. GCI was modeled as a g-factor model with one estimated residual correlation that matched theoretical considerations.

#### 2.2.4. Sustainable behavior.

Sustainable behavior was assessed with nine items (e.g., “I take a short shower instead of a bath to save water.”) in samples 1–3 and with 15 items (e.g., “I take part in demonstrations”) in samples 4–7, with five items being used in all samples. The idea was to use three items per domain that matched the respective domain content. Some items were taken from Geiger et al. [22] and some were newly written for the present study. Participants responded on a five-point response scale (1 = *never*, 2 = *rarely*, 3 = *occasionally*, 4 = *often*, and 5 = *always*). In samples 1–3, one item was excluded from analyses, because it did not fit the model. The remaining eight items were modeled as a bifactor model with a g-factor for general sustainable and prosocial behavior and a nested factor for *donation and environmental behavior*. In samples 4–7, sustainable behavior was modeled as correlated factors model (F1: *reflective behavior*, F2: *sustainable consumption*, F3: *civic and social engagement*).

#### 2.2.5. Subjective knowledge and information frequency.

Participants were asked to rate their subjective knowledge about health-, nutrition-, and environment related topics and their frequency of information seeking towards these topics in samples 1–3. In samples 4–7, participants were asked about their frequency of actively looking for information about safety issues, education, prosperity, and political issues. Subjective knowledge was assessed using one subdomain-specific item each (e.g., “How would you rate your knowledge about human rights and human rights violations?”).

#### 2.2.6. Party affiliation.

Party affiliation was assessed using a single item (“Which party would you vote for if the general elections were held next Sunday?”). The response options were: 1 = *Alternative for Germany* (AfD), 2 = *Christian Democratic Union/Christian Social Union* (CDU/CSU), 3 = *The Left*, 4 = *Free Democratic Party* (FDP), 5 = *Alliance 90/The Greens* (Greens), 6 = *Social Democratic Party of Germany* (SPD), 7 = *Others/No vote*.

#### 2.2.7. Planetary Health Knowledge – item development.

Following the structure of the PHK taxonomy (Fig 1) [5], we developed single-choice items with four response options to assess PHK in all seven domains. As PHK represents a knowledge construct with objectively correct or incorrect answers, we applied multiple-choice (MC) items that were scored dichotomously (0 = incorrect, 1 = correct) for the conduction of analyses. MC items provide a high scoring objectivity and objectivity while being suitable for assessment of declarative knowledge [46]. We used four response options (one correct and three incorrect) per item since previous research suggested that four response options provide a reasonable tradeoff between guessing probability, item discrimination, and the feasibility of constructing plausible distractors [47,48].

In all domains, we developed items in a way that ensures balanced representation of all subdomains, following psychological principles of item writing [30,49]. Furthermore, we apply the predefined facet structure to maintain thematic breadth within subdomains, thereby capturing the full conceptual breadth of PHK. In line with the PHK definition which encompasses knowledge of the current state of the world with regard to individual challenges, as well as broader contexts and interactions between various challenges and systems, we systematically apply the four aspects of knowledge (Prev, Glob, Fact, Comp) in a balanced manner across all subdomains to ensure item diversity. Most items employed verbal stimulus, whereas prevalence items were formulated as numerical questions based on official statistical data (from, e.g., World Bank, WHO, UNICEF). Each item offered four response options presented in ascending numerical order, with the correct answer evenly distributed across all response options to prevent response patterns. For global distribution respondents were asked to identify where specific problems are the most prevalent, referring to continents, individual countries, or predefined groups of three countries. In domains P1 *Health*, P2 N*utrition*, and P3 *Environment*, we also used color-coded maps, which were created with mapchart.net, as response options.

During item development, far more items were developed than ultimately used in psychometric studies. In this phase, we selected items for inclusion in the study as follows: First, all items were extensively checked for veridicality, excluding items that failed the veridicality check. Afterward, FR together with five student assistants evaluated the items’ suitability and representativeness for the study, selecting items in a way that all aspects and domains of PHK were represented in a balanced manner. In total, we tested *N* = 503 items (P1 = 60, P2 = 60, P3 = 60, P4 = 82, P5 = 81, P6 = 81, P7 = 79). See S1 Table for the initial number of items in each subdomain and item type.

### 2.3. Analytic approach

The main goals of data analyses were twofold. The first main goal was the compilation of PHK items into reduced item sets for future use. Items were removed from further consideration if they i) showed difficulties below guessing probability and ii) did not load substantially on a general factor for the respective item set. We investigated descriptive statistics and tetrachoric correlations, followed by Confirmatory Factor Analyses in which we maximized main loadings. The second main goal concerned validity issues (i.e., the nomological net), which we investigated by studying relations with gc, GCI, self-reported sustainable behavior, subjective knowledge, and party affiliation.

#### 2.3.1. Item selection and measurement models.

The selection rational was threefold: First, all items with difficulties below guessing probability (.25 due to four response options) were removed, because items below guessing probability tend to be less reliable [50]. We decided for not cutting items in the upper difficulty level as in samples with lower knowledge levels, easy items are crucial.

Second, the remaining items were modeled as a confirmatory g-factor model, assuming that items of each PHK domain reflect a common latent knowledge factor. Item selection took place using the Maximize Main Loadings (MML) approach [51], that is designed to retain items with strongest loadings on the target construct.

Items were excluded in two subsequent steps: First, those items with negative loadings were removed. Second, items with loadings below .20 on the g-factor were excluded. This cutoff is comparatively liberal. However, broad declarative knowledge domains are characterized by substantial heterogeneity in terms of thematic content, especially at higher levels of granularity of assessed knowledge by zooming more into specific topics. This often results in a certain fuzziness of the constructs, reflected by modest factor loadings [13,28]. Thus, we also considered these comparatively low loadings acceptable as the items conceptually targeted the respective PHK domain. If additional items with insufficient loadings emerged afterward, those items were iteratively deleted until only items with adequate factor loadings remained (see Fig 2 for a visualization of the item selection process).

**Fig 2:**
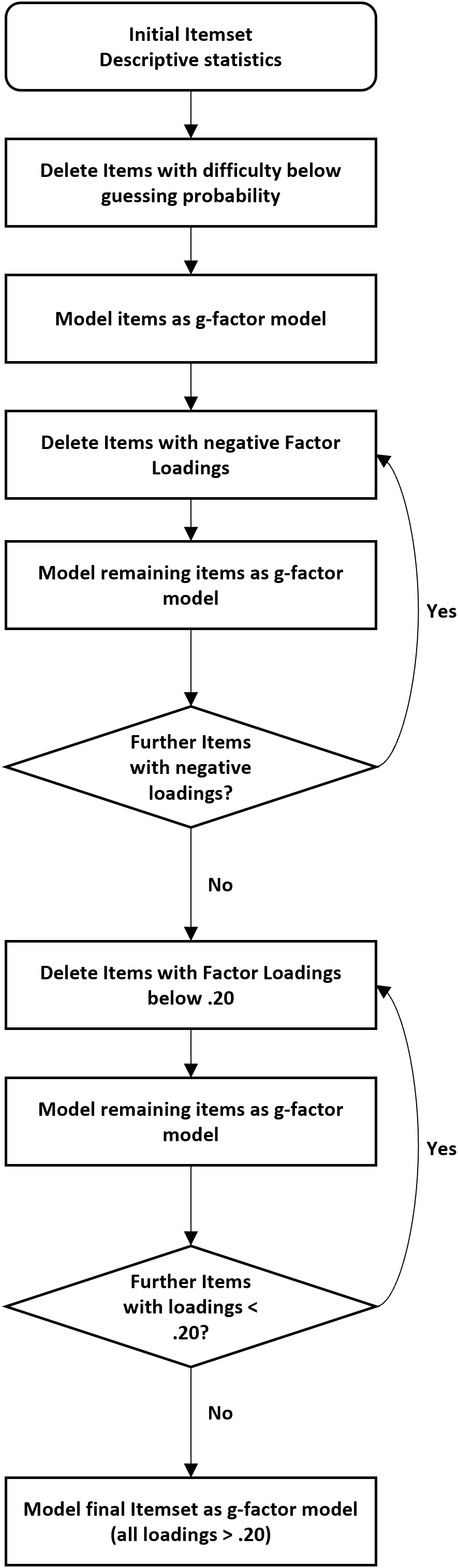
Flow Chart of Item Selection Process.

#### 2.3.2. Validity snapshot.

After establishing measurement models for each PHK domain, we estimated the correlations between each PHK factor and general knowledge, global citizenship, and sustainable behavior using CFA. Subsequently, we computed standardized mean differences across educational levels and estimated regression models in which each PHK factor was regressed on dummy-coded education variables, with middle education serving as reference group. Analogously, party affiliation was examined by comparing the preference for a specific party with the average performance of all other party preferences. For this purpose, we regressed the final PHK models on dummy-coded party variables. Due to small sample sizes (Table 1), we grouped FDP voters with CDU/CSU voters and Left voters with Others and non-voters.

#### 2.3.3. Conduction of analyses.

We used weighted least square mean and variance adjusted (WLSMV) estimators for factor analyses with binary data and robust maximum likelihood (MLR) estimators for factor analyses with continuous data. Factors were identified using the effects-coding method [52], and factor saturation was evaluated using McDonalds Omega [53]. We report scaled goodness of fit statistics. Model fit is deemed acceptable with *CFI* and *TLI* ≥ .90, *RMSEA* ≤ .08 and *SRMR* ≤ .11, and good with *CFI* and *TLI* ≥ .95, *RMSEA* ≤ .05 and *SRMR* ≤ .08 [54–56]. Additionally, we report Direct Discrepancy Dynamic Fit Index (3DFI) and use cutoffs for mediocre fit that 1corresponds to a mean absolute discrepancy (MAD) of .06 [57,58].

All analyses were conducted in *R* [version 4.4.0; 59]. Descriptive statistics were calculated using the package *psych* [version 2.4.3; 60]. CFA analyses were conducted using the package *lavaan* [version 0.6.17; 61]. The 3DFI is conducted using the package *dynamic* [version 2.4.5; 62].

**Data Availability.** We provide the complete tests and questionnaires, a summary of all analyses and supplementary analyses as well as the datasets on the Open Science Framework (OSF) (https://osf.io/8rc2q/overview?view_only=6a71499911924d15a1519dfd4afa8409).

## 3. Results

### 3.1. Psychometric analyses and item sampling

#### 3.1.1. Descriptive statistics.

All seven domain-specific initial item sets with 60–82 items were somewhat difficult with mean scores ranging from *M*_*P1*_ = .390 (*sd* = .092) to *M*_*P7*_ = .460 (*sd* = .128). Item difficulties were in a range from .017 (P4 *Safety*) to .960 (P1 *Health*) and tetrachoric correlations ranged from −.488 (P2 *Nutrition*) to .740 (P5 *Education*; Table 2).

**Table 2:** Range of item difficulties and tetrachoric correlations in initial and final item sets.

		*N Items*	Item difficulty				Tetrachoric correlations
			*Mean*	*Sd*	*Min*	*Max*	*Mean*
P1	Initial item set	60	.390	.092	.056	.960	.049
	Final item set	25	.585	.166	.314	.960	.175
P2	Initial item set	60	.421	.096	.046	.944	.044
	Final item set	25	.581	.188	.336	.944	.226
P3	Initial item set	60	.422	.105	.049	.789	.053
	Final item set	30	.544	.286	.308	.790	.194
P4	Initial item set	82	.425	.092	.017	.936	.047
	Final item set	37	.605	.170	.328	.936	.195
P5	Initial item set	81	.392	.084	.027	.834	.030
	Final item set	36	.564	.182	.314	.834	.205
P6	Initial item set	81	.428	.088	.037	.947	.044
	Final item set	36	.587	.164	.327	.917	.167
P7	Initial item set	79	.460	.128	.040	.898	.101
	Final item set	46	.588	.194	.324	.898	.253

P1 = Health, P2 = Nutrition, P3 = Environment, P4 = Safety, P5 = Education, P6 = Standard of Living, P7 = Political & Economic Systems.

Detailed information on the difficulties of domain specific item sets is provided in the supplement (https://osf.io/8rc2q/). Items with difficulties where the confidence interval included guessing probability were excluded from further analyses: P1 *Health* 26 items excluded, 34 items remaining; P2 *Nutrition* 19 items excluded, 41 items remaining; P3 *Environment* 15 items excluded, 45 items remaining; P4 *Safety 26* items excluded, 56 items remaining; P5 *Education* 29 items excluded, 52 items remaining; P6 *Standard of Living* 30 items excluded, 51 items remaining; P7 *Political & Economic Systems* 21 items excluded, 58 items remaining.

#### 3.1.2. Item sampling.

For each domain, the remaining items were modeled as confirmatory g-factor model. Except for P5 Education, all models showed an at least acceptable fit. Subsequently, items with i) negative loadings or ii) loadings below .20 were excluded. The resulting final item sets consisted of 25 (P1 Health and P2 Nutrition), 30 (P3 Environment), 37 (P4 Safety), 36 (P5 Education and P6 Standard of Living), and 47 (P7 Political & Economic Systems) items (S1 Table). In line with the item selection approach, the final item sets only involved items with difficulties above guessing probability (Fig 3). These item sets were, on average, easier—as reflected by higher mean scores—and exhibited increased tetrachoric correlations, indicating improved internal consistency (Table 2). All final model fits were acceptable or good according to traditional cutoff criteria, with at least mediocre fit according to the dynamic fit index. Specifically, for P3 Environment, the 3DFI indicated a consistent fit, showing a MAD of .00. Table 3 presents detailed model-fit indices, 3DFI cutoffs for mediocre fits, and factor saturations of all domain-specific models.

**Table 3:** Goodness of fit indices for PHK models during item sampling in studies 1 to 7.

										3DFI cutoff (mediocre fit)		
Domain	Model	N (Items)	Χ^2^	*df*	*p*	*CFI*	*TLI*	*RMSEA*	*SRMR*	*CFI*	*RMSEA*	Ω
P1	Model 1	34	577	527	.064	.915	.910	.018	.094			.677
	Model 2	32	512	464	.060	.921	.915	.019	.094			.695
	Final	25	314	275	.053	.941	.936	.022	.093	.924	.028	.740
P2	Model 1	41	868	779	.014	.920	.916	.019	.092			.709
	Model 2	35	634	560	.016	.936	.932	.021	.091			.767
	Final	25	324	275	.022	.961	.957	.024	.087	.956	.028	.807
P3	Model 1	45	982	945	.197	.973	.971	.011	.082			.722
	Model 2	38	707	665	.128	.971	.969	.014	.081			.795
	Final	30	415	405	.351	.993	.993	.009	.075	.924	.033	.819
P4	Model 1	56	1603	1484	.016	.920	.917	.016	.094			.765
	Model 2	51	1341	1224	.010	.923	.919	.018	.094			.815
	Final	37	696	629	.032	.959	.957	.019	.091	.925	.029	.838
P5	Model 1	52	1469	1274	.000	.895	.891	.023	.096			.777
	Model 2	44	1076	902	.000	.911	.906	.026	.095			.834
	Final	36	717	594	.000	.941	.937	.027	.091	.932	.030	.854
P6	Model 1	51	1291	1224	.090	.932	.929	.014	.093			.732
	Model 2	44	967	902	.066	.938	.935	.016	.093			.792
	Final	36	681	594	.007	.923	.919	.022	.096	.897	.036	.814
P7	Model 1	58	1763	1595	.002	.950	.948	.020	.094			.890
	Model 2	57	1701	1539	.002	.952	.951	.020	.093			.896
	Model 3	47	1182	1034	.001	.960	.958	.023	.092			.915
	Final	46	1127	989	.001	.963	.961	.023	.091	.957	.027	.916

P1 = Health, P2 = Nutrition, P3 = Environment, P4 = Safety, P5 = Education, P6 = Standard of Living, P7 = Political & Economic Systems. Before modeling the items as a g-factor model, we excluded items with difficulties below guessing probability. According to the dynamic fit index, a mediocre fit corresponds to a mean absolute discrepancy (MAD) of .06.

**Fig 3:**
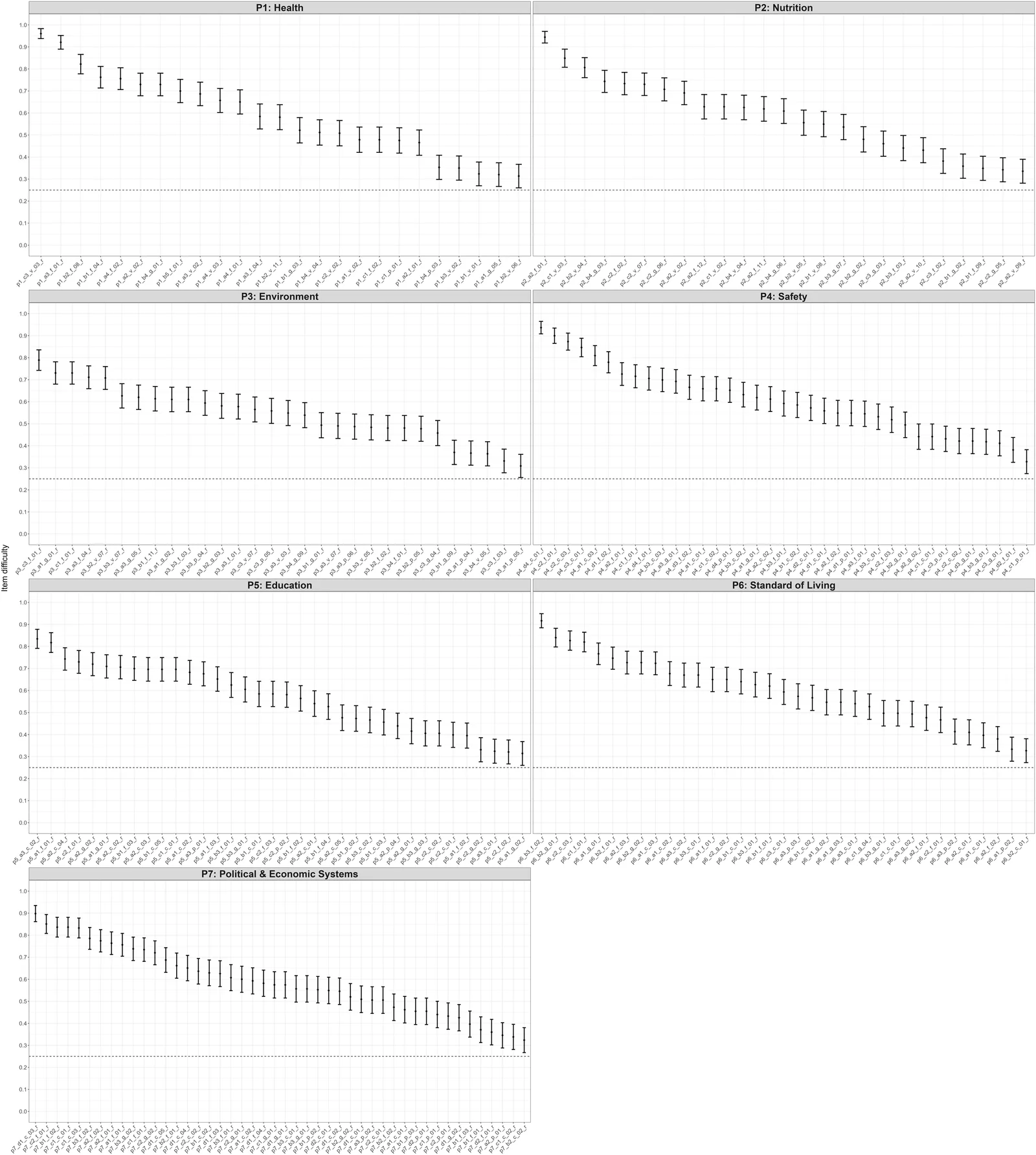
Item difficulties of final PHK Item Sets. The dashed line shows the guessing difficulty (difficulty of .25). P1 = Health, P2 = Nutrition, P3 = Environment, P4 = Safety, P5 = Education, P6 = Standard of Living, P7 = Political & Economic Systems.

All subdomains were retained and sufficiently covered in all of the final item sets, though their representation was not fully balanced. Within P1 *Health*, “Mental Health” was less strongly represented than “Health Management” and “Physical Health”, in P2 *Nutrition*,  “Sustainable Food Production” appeared most frequently, whereas in P3 *Environment*. “Climate Action” was less represented than “Environmental Change” and “Nature & Nature Conservation”. Similarly, “Everyday Safety” in P4 *Safety*, “Academic and Vocational Education” in P5 *Education*, “Mobility” in P6 *Standard of Living*, and “Participation” in P7 *Political & Economic Systems* were less represented (S1 Table). The items of the final PHK test can be found in the supporting information and on the OSF (https://osf.io/8rc2q/).

### 3.2. Validity snapshot

#### 3.2.1. PHK and gc.

Across all samples, the gc models fit the data well according to traditional cutoffs and reaches mediocre fit or better according to dynamic fit (see S2 Table) and factor saturations were high (Ω > .70). Across the different samples, gc-mean scores ranged from .53 (P1 *Health*) to .58 (P5 *Education* and P7 *Political & Economic Systems*). Bivariate latent correlations with individual PHK domains were lowest for P7 *Political & Economic Systems* (*r* = .539, *p* < .001) and highest for P3 *Environment* (*r* = .761, *p* < .001; Table 4).

**Table 4:** Bivariate latent correlations for PHK domains with covariates.

		P1	P2	P3	P4	P5	P6	P7
GC	g	**.736**	**.733**	**.761**	**.719**	**.577**	**.620**	**.539**
GCI	g	**.272**	**.238**	**.319**	**.365**	**.359**	**.315**	**.416**
Behavior	g	**.278**	**.246**	**.231**	**–**	–	–	–
	nf1	**-.370**	**-.444**	**-.295**	**–**	–	–	–
	F1	–	–	–	**.255**	**.203**	-.033	.132
	F2	–	–	–	**.578**	**.305**	.134	**.406**
	F3	–	–	–	**-.161**	**-.179**	**-.236**	**-.146**
Subjective Knowledge	g	.045	.115	.112	**.133**	**-.206**	.106	**.240**
Information frequency	g	.069	.113	.149	**.184**	.127	.073	**.222**

The same items were used to assess behavior in domains P1, P2, and P3, and in domains P4, P5, P6, and P7, respectively. P1 = Health, P2 = Nutrition, P3 = Environment, P4 = Safety, P5 = Education, P6 = Standard of Living, P7 = Political & Economic Systems. GC = Crystallized intelligence (general knowledge), GCI = Global Citizenship Identification. Behavior: g = g-factor, nf1 = nested factor (donation behavior), F1 = Sustainable consumption, F2 = Economic and social reflective behavior, F3 = Civic and social engagement. Values in **bold** indicate values are significant at an alpha level of .05.

#### 3.2.2. PHK and GCI.

GCI models fit the data mostly acceptably to well across all seven samples (see S2 Table). The GCI models had high factor saturations (Ω > .70). Across all PHK domains, GCI and PHK were – as expected – low to moderately correlated with each other. We found lowest correlations for P2 *Nutrition* (*r* = .238, *p* < .001) and highest correlations for P7 *Political & Economic Systems* (*r* = .416, *p* < .001; Table 4).

#### 3.2.3. PHK and behavior.

The bifactor models for behavior had mostly acceptable fit in domains P1, P2, and P3 (S2 Table). Factor saturations were moderate for the g-factor (Ω_g_ = .510 to .582) and low for the nested factor (Ω_donation_ = .326 to .331). General sustainable and prosocial behavior was weakly positively correlated with PHK domains P1 *Health*, P2 *Nutrition*, and P3 *Environment* (*r* = .231 to .278). Beyond this, the nested factor for donation and environmental behavior was negatively correlated with those three domains (*r* = − .246 to −.370; Table 4).

In domains P4 to P7, behavior was modeled as three correlated factors. These models fit the data mostly acceptably and factor saturations were moderate for the factor economic and social reflective behavior (Ω = .458 to .538) and high for the factor sustainable consumption (Ω = .606 to .709) and the factor civic and social engagement (Ω = .753 to .776). PHK correlated weakly positive with sustainable consumption, but only the correction with P4 *Safety* was inferentially larger than zero (*r* = .255, *p* = .001) and descriptively but not inferentially weakly negative with civic and social engagement (*r* = −.146 to −.236, all n.s.). Correlations of PHK domains were highest with economic and social reflective behavior *r*_*P6*_ = .134, *p* = .085 to *r*_*P4*_ = .578, *p* < .001; Table 4).

#### 3.2.4. PHK and education.

Across all domains participants with higher education levels reached higher scores in PHK than participants with middle and low education (Fig 4). Regressing PHK on dummy coded education variables indicated that participants with low educational attainment were significantly less knowledgeable than participants with high or middle educational attainment in the domains P1, P2, and P7 (β_P1_ = −.668, β_P2_ = −.640, β_P7_ = −.460). Further, participants with high educational attainment were significantly more knowledgeable than participants with low or middle educational attainment in the domains P2, P3, and P4 (β_P2_ = .449, β_P3_ = .471, β_P4_ = .689; Table 5). In total, educational attainment explained between 5.70% (P5) and 20.2% (P2) of the variance in PHK scores.

**Table 5:** Standardized latent regression coefficients for PHK with subjective knowledge & information frequency, education, and party affiliation.

		P1	P2	P3	P4	P5	P6	P7
Information frequency		.083	.065	.152	.188	**.339**	-.004	.161
Subjective knowledge		-.015	.070	-.006	-.012	**-.401**	.109	.131
Education	Low vs. Middle/High	**-.663**	**-.640**	-.261	-.039	-.260	**-.317**	**-.460**
	High vs. Middle/Low	.232	**.449**	**.471**	**.689**	**.333**	.284	.223
Political Party	AfD	-.300	.023	**-.379**	-.289	**-.423**	-.178	**-.406**
	FDP/CDU/CSU	-.221	-.109	.068	.077	.028	-.138	.036
	Greens	**.606**	**.703**	**1.011**	**.589**	.380	**.729**	**.715**
	SPD	.141	-.269	-.076	-.273	.134	-.119	**.468**
	Others/ No vote/ Left	-.097	-.091	-.100	.063	.056	.039	**-.343**

Education: Low = lower secondary degree or vocational track, Middle = middle secondary degree or intermediate track, High = higher secondary degree or academic track, AfD = Alternative for Germany (far-right party), CDU /CSU = Christian Democratic/Social Union (center-right conservative party), FDP = Free Democratic Party, SPD = Social Democratic Party of Germany (social democratic party), Left = The Left (left party), Greens = Alliance 90/The Greens (ecological party). Due to small sample size in some party groups, we grouped the FDP with CDU/CSU, and the Left with Others and Non-voters. Values in **bold** indicate values are significant at an alpha level of .05.

**Fig 4:**
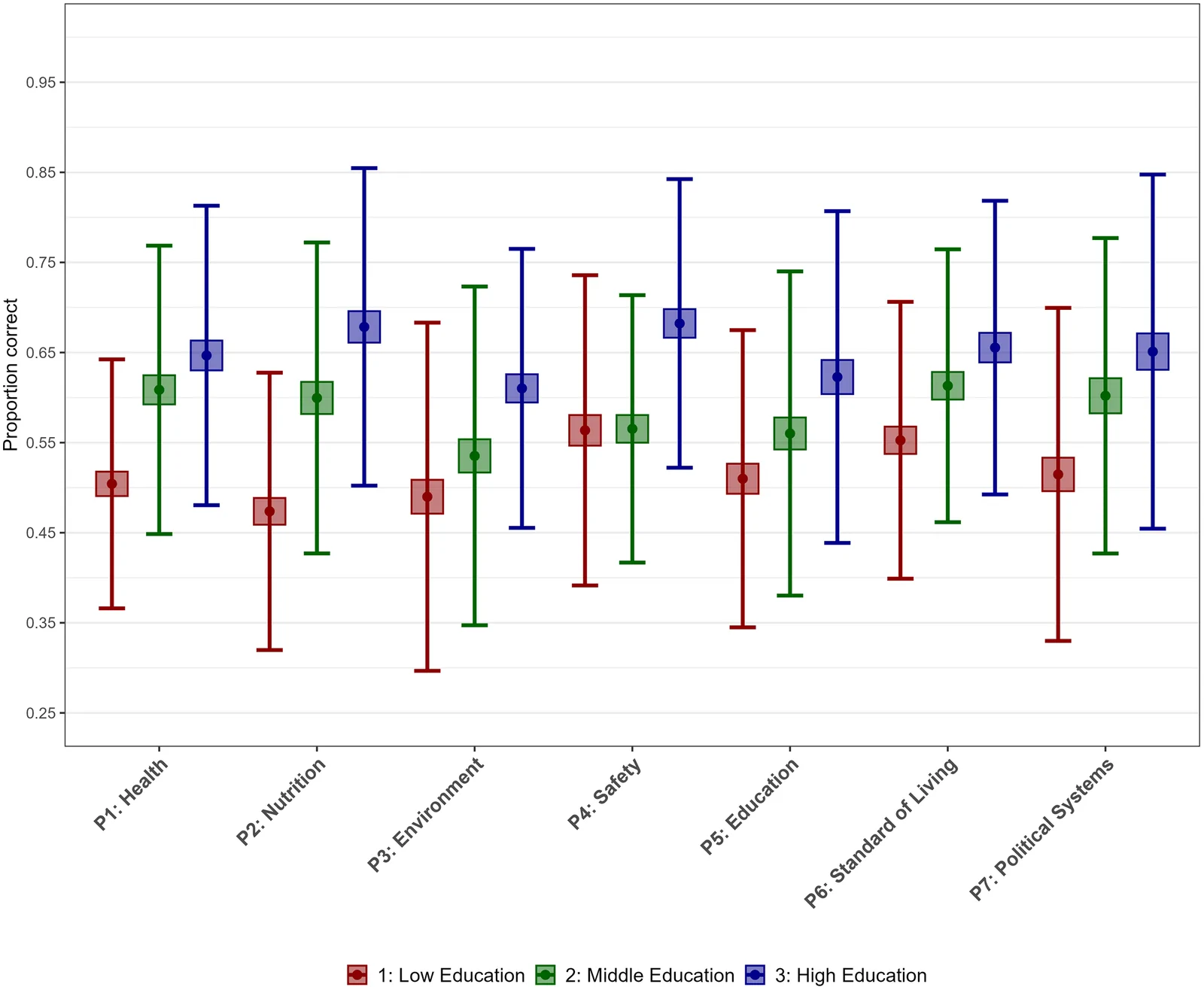
Mean Levels of Planetary Health Knowledge by Level of Education. Due to the block design participants and sample sizes per party group varied across the domains. Boxes represent the range of the mean + /- 1 SE; whiskers represent the range of the mean + /- 1 SD. Low Education = lower secondary degree or vocational track, Middle Education = middle secondary degree or intermediate track, High Education = higher secondary degree or academic track.

#### 3.2.5. PHK and subjective knowledge and information frequency.

Investigating the latent bivariate correlations between all PHK domains on one side and subjective knowledge and the frequency of information seeking on the other side revealed mostly positive, weak, and nonsignificant correlations (Table 4). The strongest correlations were observed for P7 *Political & Economic Systems* (*r*_*Knw*_ = .240, *p* < .001; *r*_*Info*_ = .222, *p* = .001). In contrast, P5 *Education* showed a negative correlation with subjective knowledge.

When regressing the PHK domains on information-seeking frequency and subjective knowledge in a multivariate regression model, neither information-seeking frequency (β_Info_ = −.004 to .161) nor subjective knowledge (β_Knw_ = −.015 to .131) was significantly predictive of PHK in six of seven PHK domains (Table 5). Only for P5 *Education*, information-seeking frequency was found to positively predict PHK (β_Info_ = .339, *p* < .001), while subjective knowledge emerged as a negative predictor (β_knw_ = −.401, *p* < .001).

#### 3.2.6. PHK and party affiliation.

Analyses of the mean performance in PHK across party groups revealed voters of the ecological party (the Greens) outperformed voters of other parties across all seven PHK domains (Fig 5). Additionally, voters of the far-right populist AfD received lowest scores in most domains, especially in P3 *Environment*, P4 *Safety*, P5 *Education*, and P7 *Political & Economic Systems*.

**Fig 5:**
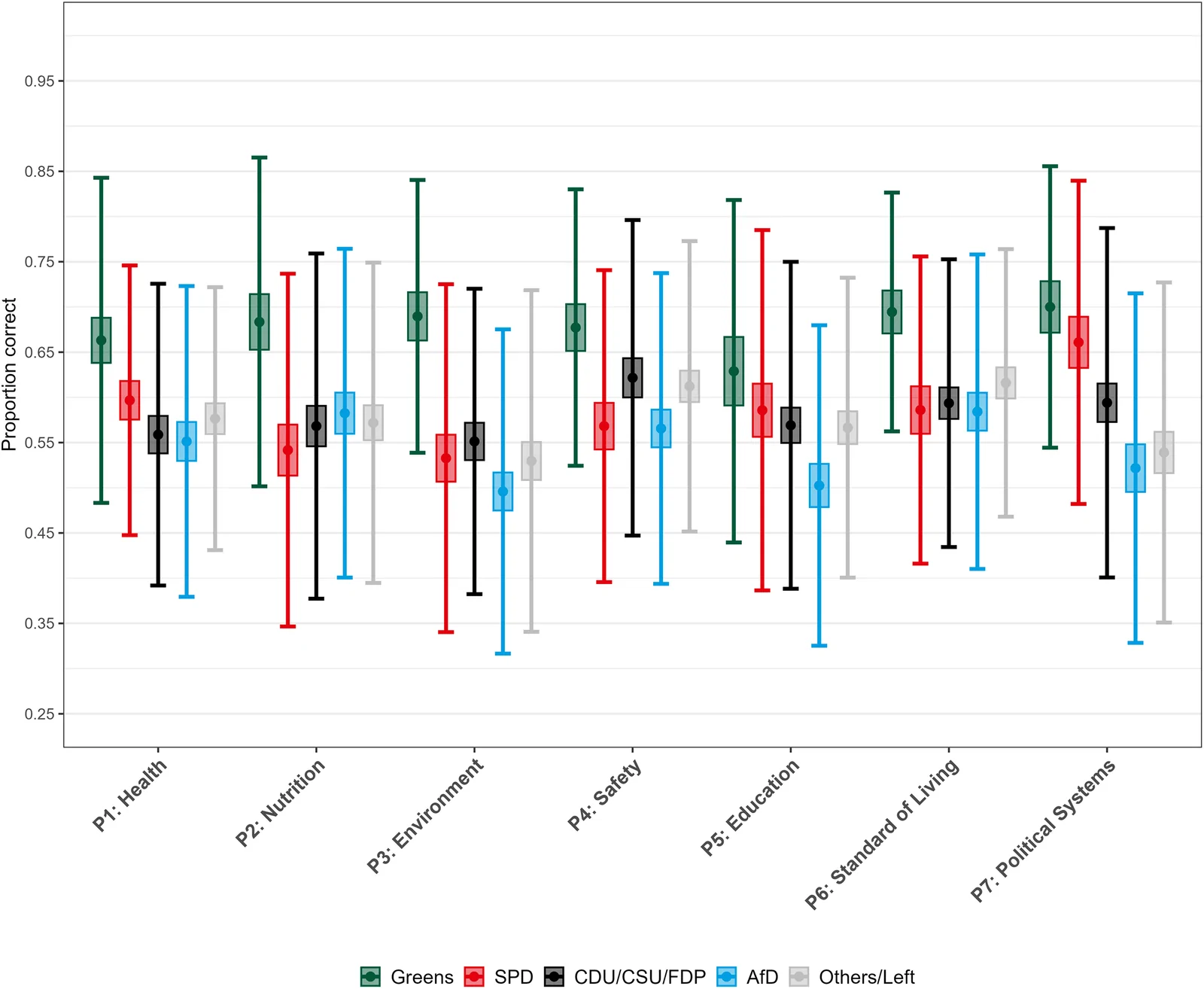
Mean Levels of Planetary Health Knowledge by Party Affiliation. Due to the block design participants and sample sizes per party group varied across the domains. Boxes represent the range of *M* + /- 1 *SE*; whiskers represent the range of *M* + /- 1 *SD*. AfD = Alternative for Germany (far-right party), CDU /CSU = Christian Democratic/Social Union (center-right conservative party), FDP = Free Democratic Party, SPD = Social Democratic Party of Germany (social democratic party), Greens = Alliance 90/The Greens (ecological party), Others = others (including the left) and non-voters. Voters of the FDP were grouped with the CDU/CSU and voters of the left were grouped with others due to small sample size.

Regression analyses confirmed these patterns: except for P5 *Education*, voters of the Greens scored between 0.585 and 1.011sd higher than voters of other parties (Table 5). Conversely, AfD voters scored 0.379 sd lower in P3 *Environment* and 0.423 sd lower in P5 *Education*, while non-voters and voters of small parties scored 0.406 sd lower in P7 *Political & Economic Systems* compared to voters of other parties (Table 5).

## 4. Discussion

We first summarize key findings and then address implications for the understanding of *Planetary Health Knowledge* (PHK). The PHK item pool was built upon and derived from a PHK taxonomy [5]. Data were collected in seven domain-specific blocks in which we investigated psychometric quality and relations with covariates.

### 4.1. Key findings

Across all domains, initial item difficulties were high with an average of 39–46% correct solutions across blocks. Difficulties were particularly high amongst items asking about prevalence or global distribution. Item selection was geared towards retaining items suitable for use in general population samples. Selection procedures adhered to a) appropriate difficulty as indicated in solution probabilities above guessing probability and below unity and b) coherence as indicated in positive factor loadings of non-trivial magnitude. As a result of this selection process, many of the prevalence and global distribution items were dropped from further analyses.

Associations with concurrent variables revealed moderate to high correlations with general knowledge (gc) across all PHK domains. Associations were strongest for P3 *Environment* and lowest for P7 *Political & Economic Systems.* Expectedly, all PHK domains were moderately correlated with *Global Citizenship Identification* (GCI). Correlation with GCI was highest for P7 *Political & Economic Systems* domain and lowest for P2 *Nutrition*, presumably reflecting expected content overlap.

Concerning behavioral aspects, PHK correlated expectedly weak to moderate with sustainable and prosocial behaviors across domains. These findings are also consistent with previous research [6,22,33]. This supports the notion that the knowledge-behavior link is mediated by intentions and moral norms [6]. Interestingly, in domains *Health*, *Nutrition*, and *Environment*—where behavior was modeled as bifactor model—the nested donation behavior factor was negatively correlated with PHK. This suggests that individuals who generally report more prosocial and environmentally responsible behavior may engage less in donation behavior once their overall sustainability tendencies are accounted for. In the remaining four domains, PHK was negatively correlated with civic and social engagement, indicating that individuals with higher PHK levels tend to participate less in political and civic actions. Further, PHK correlated moderately with economic and social reflective behavior, implying that more knowledgeable individuals weigh financial resources more carefully and deliberate longer before making consumption decisions. Last, we found only weak correlations with sustainable consumption behavior.

In line with previous findings and our hypothesis, subjective knowledge and information-seeking frequency showed only weak associations with PHK. Consistent with this, the multivariate regression analyses indicated that except for P5 *Education*, both variables significantly predicted PHK. This underscores the limited overlap between self-reported indicators and factual knowledge.

Unsurprisingly, all PHK domains were strongly associated with educational attainment, consistent with prior evidence linking years of education to knowledge [39,63,64]. Formal education represents a central environmental factor in cognitive development and is associated with increases in broad cognitive abilities [39]. Clearly, PHK represents aspects of crystallized intelligence (gc), the breadth and depth of acquired knowledge [e.g., 65]. Just like other aspects of gc, PHK is fostered by good learning opportunities, intellectual interest, a sufficient amount of learning time and fluid intelligence [13,66]. PHK represents a compilation of knowledge from several domains and because it is due to the same limiting factors and similar beneficial conditions, it correlates strongly with related knowledge. Further, prior knowledge facilitates further acquisition of new [67–69]. Motivational factors—including interests—also contribute to knowledge acquisition and may mediate the effects of prior knowledge on learning outcomes [70–72]. Interests, in particular, are positively associated with the engagement and knowledge development [70]. Finally, quantity and quality of formal and informal learning opportunities strongly influence knowledge accumulation, and such opportunities vary systematically across educational pathways [39].

Associations of political preferences (as measured through voting preference for political parties) with PHK, Green Party voters score higher than voters of other parties, across all domains, whereas supporters of the far-right populist *Alternative for Germany* (AfD) were less knowledgeable across domains. These differences are presumably largely attributable to disparities in educational attainment and the effects substantially diminish when controlling for education.

### 4.2. Outlook

The present findings indicate that PHK and gc are somewhat different. Correlations were somewhat smaller than results reported in previous studies [22]. However, this does not imply that general knowledge and PHK rely on fundamentally different cognitive constructs. In both cases, the underlying construct can be understood as declarative knowledge or gc, while differences primarily lie in the thematic scope of the assessed knowledge. Associations with gc were strongest for the *Environment* and lowest for the *Political & Economic Systems* domains. These differences may have several underlying causes. One possible cause is that domains differ in the extent to which knowledge acquisition adheres to the same mechanisms that are in charge of acquiring knowledge in formalized settings of learning, i.e., school. Domains in which learning is supposedly driven more strongly by personal interests may show weaker correlations with gc, and instead might be more strongly related with subjective knowledge or other self-report measures of interest and preferences. Additionally, of all PHK Dimensions *Political & Economic Systems* domain shows the strongest relation with GCI. This is also the domain which correlates weakest with gc. This might in turn indicate that interests or identification can be a more important driver of knowledge acquisition in some domains than in others, but can be expected to show a weak positive relation on average.

Furthermore, political or ideological attitudes might be a driver of differences in PHK. Regarding differences in PHK scores across political parties, a first inspection reveals such mean differences. However, once we examine the distribution of party affiliation and educational attainment, it becomes apparent that particularly Green Party voters have higher levels of education than voters of other parties, especially those voting for the AfD. Once these are controlled for, the relations between party affiliation and PHK is substantially attenuated. Although the present correlative design does not easily allow for causal conclusions, one may wonder whether individuals vote for specific parties because they lack an understanding of complex issues. If this is the case, there is an even greater need to increase the education on complex issues and global and environmental relations especially for lower educational levels.

Although knowledge is widely regarded essential for effective environmental interventions, our findings clearly speak against this position of philosophical enlightenment and echo the grim empirical reality that merely knowing sobering facts does not automatically translate into behavior change. Nonetheless, changes in environmental knowledge as well as PHK may still be deemed an end in itself, particularly when interventions aim to foster responsible citizenship.

### 4.3. Implications

PHK tests may provide several applications for both, research and educational contexts. The instrument is primarily intended to be used as a research tool to assess and track PHK, to investigate relations to other relevant variables, and to examine changes in knowledge over time. Further, the taxonomy-based structure of PHK could be used to support the development and evaluation of educational interventions. As discussed in global competence frameworks and large-scale assessments [9,73], knowledge about global interrelations and issues is required to engage in addressing global issues like human rights violations, environmental issues, or violence. As such, the PHK framework could be used to implement teaching units in which students learn about single issues and their interrelations. Promoting such teaching units and fostering the implementation in curricula may also increase the level of engagement for addressing global issues.

Importantly, the current tests are developed to serve as research instruments. They are not, yet, intended for individual diagnostics but rather for research or educational evaluation. Also, the items have been developed for adult target populations. The use in specific age groups might require adaptations and the development of additional items with appropriate levels of difficulty and content. Prevalence items—numerical, data-based items—were the most difficult indicators. Participants tended to select the most negative response options. Indeed, prior research found people often misjudge the state of the world, tending to predominantly pessimistic estimations of global conditions due to a negativity bias [74]. One possible explanation is that respondents rely on simple heuristics when making prevalence estimations [75], a process that may also apply to estimates of global and environmental issues and problems. If responses are driven more by heuristic processing than by stored factual knowledge, this begs the question about the suitability of such items for assessing crystallized intelligence. Conventional gc tests also use numerical stimuli, but mostly these items do not trigger worries about the fate of the planet that are then expressed by some numerical estimate. Taken together prevalence items can capture crystallized knowledge but they are vulnerable to effects from worldviews and cognitive biases. Nevertheless, knowledge about frequencies, magnitudes and the like are an intrinsic part of PHK. Although, reproduction of frequencies, magnitudes, or data is per se not decisive, such knowledge is a prerequisite to develop justified and sound ideas about the scope of a problem and how for instance to evaluate effects of interventions. Please note, that *prevalence* and *global distribution* items are indexically tied to time and place and additionally exhibit temporal dependency. Therefore, these items require more regular updates to maintain actuality than other sections (which of course also need maintenance).

Importantly, item and test difficulty were part of the item sampling process. By eliminating items below guessing probability and maximizing factor loadings, items were selected in a way that difficulties and factor loadings were appropriate for educationally heterogenous samples. Given that individuals with higher education levels achieve higher test scores than those with lower education, item compilation might need adjustment in either highly or lowly educated samples. More sophisticated item sampling techniques such as Ant Colony Optimization (ACO) can be used to optimize several selection criteria (such as factor loadings, factor saturation, mean difficulty, correlations with external criteria, and construct coverage) at the same time [28,76].

In the broader field, subjective knowledge is often conflated with subjective factual knowledge, although both represent distinct constructs. PHK correlated moderately with global citizenship identification (GCI) across all domains, consistent with the notion that global awareness and knowledge are core elements of global citizenship [4,77]. However, recent findings suggest that *subjective* knowledge may be an even stronger predictor of GCI than *objective* knowledge [19]. This is problematic for two reasons: First, subjective and factual knowledge are only weakly related—as demonstrated in the present study and supported by recent research [35,78]. Second, they reflect different dispositions: Subjective knowledge reflects typical behavior, self-perception or attitudes, whereas objective knowledge represents an ability, and abilities require measurement tools consistent with the logic of ability treating subjective knowledge as a proxy for objective knowledge therefore risks the development of Jingle fallacies [79,80].

For those reasons, PHK should be assessed using ability-based, performance-oriented measures rather than self-report. Relying on self-reported knowledge not only risks construct contamination but may also artificially inflate correlations with GCI. It is important to emphasize that correlations among ability measures tend to be substantially higher than those between ability and self-report measures, further underscoring the need for ability-based assessment of knowledge.

Beyond the psychometric properties of the final scales, it is important to consider the content coverage of the final item sets. With respect to content breadth, all theoretically specified subdomains were still represented in the final item sets, indicating that construct coverage was retained even after data-driven item selection. Regarding the methodological composition, the distribution of knowledge aspects (Prevalence, Global distribution, Declarative knowledge, Comprehension) became somewhat less balanced in the final item pools. Prevalence and Global Distribution items were retained less frequently than Declarative Knowledge and Comprehension items.

From a substantive perspective, environmental knowledge, sustainability knowledge, and global citizen knowledge can be understood as integral components of PHK (see Rees & Wilhelm [5], for a comparison of sustainability, global citizenship, and planetary health). As such, the PHK test lends itself to a broad range of applications. Depending on the research question, PHK may be used either as an overall construct or at the level of specific domains.

### 4.4. Limitations and implications for further research

One limitation of this study is its block design, which restricted PHK assessment on the domain level to seven separate domains. Although the block design allowed for inclusion of a substantial number of knowledge items *within* each PHK domain, it precluded analysis of the covariance structure *across* domains. Hence, a sizable number of knowledge items was available per domain but the present design does not allow to study the covariance structure across domains. A comprehensive study integrating the seven domains is pending and therefore we cannot yet establish an overarching second-order measurement model for PHK.

In the current studies, we used the same sample for both, item sampling and model evaluation. Consequently, some degree of overfitting cannot be ruled out. A split-sample cross-validation approach was not feasible given the available sample size. Future studies should therefore replicate and cross-validate the present findings using independent samples. Another limitation concerns the sampling approach based on online panel recruitment. Although we applied quota sampling to approximate population characteristics, online panels are susceptible to selection biases. Particularly, participants register for survey panels and are willing to participate in online surveys [81]. Previous research found that older age, lower educational status, and lower survey participation willingness are underrepresented in such panels [81,82]. Quota-based recruiting can partially correct for such biases, but this cannot completely eliminate differences that may occur between participants that are registered in online panels and such who are not. The generalizability of such results might therefore be deemed somewhat limited.

Further, across all PHK domains, correlations with gc were smaller than in previous studies. This might reflect restrictions in nomological breadth of the PHK domains or a lack of fidelity and coverage in the gc measure. In each sample, PHK covered a single domain and therefore, did not capture the full breadth of PHK, which spans across traditional gc domains (natural sciences, social sciences, humanities, life sciences). Consequently, the present findings may underestimate the associations that would emerge in a more multivariate within-subject study with comprehensive, cross-domain measure of PHK. Similarly, gc was assessed using a short measure, which inherently entails limited content coverage, reliability, and validity. Moreover, PHK’s strong focus on current global conditions—effectively a form of current events knowledge—and the inclusion of prevalence items and questions about global distributions may have attenuated the correlations with gc.

Another limitation might be that compilation of items across all abbreviated forms still results in a rather lengthy test with very broad coverage. A key goal of the present study was to provide tools to the scientific community, future work should further refine the item pool to allow for further abbreviation. Preferably such research would also provide parallel forms, to permit using the tests readily in intervention studies, for example.

Further, factors such as socio-economic status, interest, information behavior, and learning opportunities (e.g., travel, personal affectedness, quality of instruction) shape knowledge acquisition. Individuals directly affected by global issues—such as food insecurity or rising sea levels—may also develop greater domain-specific knowledge. Future research should therefore investigate how affectedness, experiences, culture, and socioeconomic status contribute to PHK.

A minor limitation of this study concerns the social desirability in self-report scales (GCI, behavior and party affiliation). We have observed a fair amount of variance in the respective scales and assume that this is not a significant concern in the current study. Regarding the party affiliation, we have observed around 20% of the participants would have voted for the AfD, which, given its right-wing populist position, would presumably be the least socially desirable response. The observed 20% AfD voters corresponds to survey results from the respective time period, when the AfD reached new records with 20% [83,84]. One concern with specific social desirability scales is that they have been questioned in the methodological literature as they are likely to assess not only response biases, but also substantial personality variance [85]. Nevertheless, future studies may investigate the respective effects while controlling for social desirability.

Last, there are many researcher degrees of freedom to group party affiliation. Due to limited sample size for this purpose, smaller parties were not considered individually. Instead, we decided to group them with nearby parties or Others/non-voters. Specifically, the FDP was grouped with the CDU/CSU, whereas the Left was grouped with Others/non-voters. Interpretation of these effects must consider these decisions. To provide greater transparency, we added a table to the Appendix reporting manifest PHK scores for the original party groups—including separate scores for FDP and Left voters (S3 Table). Future studies that oversample smaller groups might be in a position to compare knowledge of more political groups.

## 5. Conclusion

Planetary Health Knowledge (PHK) describes knowledge about the world and major global issues. We developed an extensive PHK item pool consisting of 236 items across seven PHK domains that are suitable to be used for testing in the general population. All materials are free and publicly available on OSF and in the appendix (S4 Appendix). Translations into various languages will be provided gradually. Good psychometric properties could be established for all seven PHK domains. PHK is highly correlated with general knowledge and shows moderate correlations with global citizenship identification. In line with prior research, PHK was only weakly to moderately associated with sustainable and prosocial behavior. PHK was essentially unrelated with subjective knowledge and we strongly suggest to use objective knowledge tests to measure knowledge and not subjective self-report ratings. Concerning political affiliation, voters of the ecological party were found more knowledgeable in PHK than voters of other parties, but this advantage was mostly due to these voters having higher educational attainment on average. For applied purposes future research should provide easy to use short measures. Further, investigation of the nomological network of PHK is still pending.

## Supporting information

S1 Table Number of items in initial and final item sets in seven PHK domains. (DOCX)

S2 Table Model fit indices for covariate models in studies 1–7. (DOCX)

S3 Table Manifest PHK scores for individual and grouped party groups in studies 1–7. (DOCX)

S4 Appendix PHK Test with Material.zip. (ZIP)

## References

[1] Rakowski JJ, Schaan LN, Van Klink R, Herzon I, Arth A, Hagedorn G. Characterizing the global polycrisis: a systematic review of recent literature. Annu Rev Environ Resour. 2025;50:159–83. doi: 10.1146/annurev-environ-111523-102238

[2] Jochem C, von Sommoggy J, Hornidge A-K, Schwienhorst-Stich E-M, Apfelbacher C. Planetary health literacy: A conceptual model. Front Public Health. 2023;10:980779. doi: 10.3389/fpubh.2022.980779 36726624 PMC9886088

[3] Bourke L, Bamber P, Lyons M. Global citizens: Who are they?. Education, Citizenship and Social. 2012;7(2):161–74. doi: 10.1177/1746197912440858

[4] Reysen S, Katzarska-Miller I. A model of global citizenship: antecedents and outcomes. Int J Psychol. 2013;48(5):858–70. doi: 10.1080/00207594.2012.701749 22804560

[5] Rees F, Wilhelm O. What you need to know about the world: Toward a taxonomy of planetary health knowledge. Front Public Health. 2025;13. doi: 10.3389/fpubh.2025.1564555 PMC12098363 40416652

[6] Bamberg S, Möser G. Twenty years after Hines, Hungerford, and Tomera: a new meta-analysis of psycho-social determinants of pro-environmental behaviour. J Environ Psychol. 2007;27:14–25. doi: 10.1016/j.jenvp.2006.12.002

[7] Kioupi V, Voulvoulis N. Education for Sustainable Development: A Systemic Framework for Connecting the SDGs to Educational Outcomes. Sustainability. 2019;11(21):6104. doi: 10.3390/su11216104

[8] UNESCO. Schools in action, global citizens for sustainable development: a guide for students. Paris: United Nations Educational, Scientific and Cultural Organization. 2016.

[9] OECD. PISA 2018 assessment and analytical framework. OECD Publishing. 2019. doi: 10.1787/b25efab8-en

[10] Whitmee S, Haines A, Beyrer C, Boltz F, Capon AG, de Souza Dias BF, et al. Safeguarding human health in the Anthropocene epoch: report of The Rockefeller Foundation-Lancet Commission on planetary health. Lancet. 2015;386(10007):1973–2028. doi: 10.1016/S0140-6736(15)60901-1 26188744

[11] United Nations. Transforming our world: The 2030 agenda for sustainable development. New York: United Nations General Assembly. 2015. https://documents.un.org/doc/undoc/gen/n15/291/89/pdf/n1529189.pdf?token=3Hdq2WNegvyTqgVqbY&fe=true

[12] Wilhelm O, Kyllonen P. To predict the future, consider the past: Revisiting Carroll (1993) as a guide to the future of intelligence research. Intelligence. 2021;89:101585. doi: 10.1016/j.intell.2021.101585

[13] Ackerman PL. A theory of adult intellectual development: Process, personality, interests, and knowledge. Intelligence. 1996;22(2):227–57. doi: 10.1016/s0160-2896(96)90016-1

[14] Coombs PH, Ahmed M. Attacking rural poverty. How nonformal education can help. 1974.

[15] Steger D, Schroeders U, Wilhelm O. On the dimensionality of crystallized intelligence: A smartphone-based assessment. Intelligence. 2019;72:76–85. doi: 10.1016/j.intell.2018.12.002

[16] Rosling O. Gapminder. https://www.gapminder.org/about/about-gapminder/ 2022. Accessed 2022 August 5.

[17] Barrows TS. College students’ knowledge and beliefs: a survey of global understanding: the final report of the Global Understanding Project. Global Understanding Project, Educational Testing Service, Council on Learning (U.S.) New Rochelle, N.Y: Change Magazine Press. 1981.

[18] Zwickle A, Koontz M, Slagle K, Bruskotter T. Assessing sustainability knowledge of a student population: Developing a tool to measure knowledge in the environmental, economic and social domains. Int J Sustain High Educ. 2014;15:375–89. doi: 10.1108/IJSHE-01-2013-0008

[19] Reysen S, Katzarska-Miller I, Gibson SA, Hobson B. World knowledge and global citizenship: factual and perceived world knowledge as predictors of global citizenship identification. Int J Dev Educ Glob Learn. 2013;5:49–68.

[20] Frick J, Kaiser FG, Wilson M. Environmental knowledge and conservation behavior: exploring prevalence and structure in a representative sample. Personal Individ Differ. 2004;37:1597–613. doi: 10.1016/j.paid.2004.02.015

[21] Geiger SM, Otto S, Diaz-Marin JS. A diagnostic Environmental Knowledge Scale for Latin America / Escala diagnóstica de conocimientos ambientales para Latinoamérica. PsyEcology: Bilingual Journal of Environmental Psychology. 2014;5(1):1–36. doi: 10.1080/21711976.2014.881664

[22] Geiger SM, Geiger M, Wilhelm O. Environment-specific vs. general knowledge and their role in pro-environmental behavior. Frontiers in Psychology. 2019. doi: 10.3389/fpsyg.2019.00718 PMC6454026 31001174

[23] Kaiser FG, Frick J. Entwicklung eines Messinstrumentes zur Erfassung von Umweltwissen auf der Basis des MRCML-Modells. Diagnostica. 2002;48(4):181–9. doi: 10.1026//0012-1924.48.4.181

[24] Maurer M, Bogner FX. Modelling environmental literacy with environmental knowledge, values and (reported) behaviour. Stud Educ Eval. 2020;65:100863. doi: 10.1016/j.stueduc.2020.100863

[25] Player L, Hanel PHP, Whitmarsh L, Shah P. The 19-Item Environmental Knowledge Test (EKT-19): A short, psychometrically robust measure of environmental knowledge. Heliyon. 2023;9(8):e17862. doi: 10.1016/j.heliyon.2023.e17862 37609389 PMC10440470

[26] Fauville G, Strang C, Cannady MA, Chen YF. Development of the International Ocean Literacy Survey: Measuring Knowledge Across the World. Environ Educ Res. 2019;25:238–63. doi: 10.1080/13504622.2018.1440381

[27] Beier ME, Ackerman PL. Determinants of health knowledge: An investigation of age, gender, abilities, personality, and interests. J Pers Soc Psychol. 2003;84:439–48. doi: 10.1037/0022-3514.84.2.439 12585815

[28] Steger D, Jankowsky K, Schroeders U, Wilhelm O. The Road to Hell Is Paved With Good Intentions: How Common Practices in Scale Construction Hurt Validity. Assessment. 2023;30(6):1811–24. doi: 10.1177/10731911221124846 36176178 PMC10363927

[29] Geiger M, Rees F, Santana AP, Shamrizi P, Böhm R, Wilhelm O. Measuring knowledge, attitudes, and behavior regarding antibiotics use and AMR development. In: Understanding Antibiotic Use: From the Lab to the Wild, Bremen, 2023.

[30] Clark LA, Watson D. Constructing validity: Basic issues in objective scale development. Methodological issues and strategies in clinical research (4th ed.). American Psychological Association. p. 187–203. doi: 10.1037/14805-012

[31] Loevinger J. Person And Population As Psychometric Concepts. Psychol Rev. 1965;72:143–55. doi: 10.1037/h0021704 14282672

[32] Oxfam. Education for global citizenship: A guide for schools. Oxfam Gb. 2015. https://oxfamilibrary.openrepository.com/bitstream/handle/10546/620105/edu-global-citizenship-schools-guide-091115-en.pdf?sequence=11&isAllowed=y

[33] Betsch C, Geiger M, Lehrer L, Sprengholz P, Temme H, Tiede K. Modelling the readiness for climate action across individual and system-related behaviours. OSF. 2025. doi: 10.31219/osf.io/enkwy_v3

[34] Meinhold JL, Malkus AJ. Adolescent environmental behaviors: Can knowledge, attitudes, and self-efficacy make a difference?. Environ Behav. 2005;37:511–32. doi: 10.1177/0013916504269665

[35] Goecke B, Schroeders U, Zettler I, Schipolowski S, Golle J, Wilhelm O. The Nomological Net of Knowledge, Self-Reported Knowledge, and Overclaiming in Children. J Pers Assess. 2023;105(5):702–13. doi: 10.1080/00223891.2022.2144332 36441513

[36] de Vreese CH, Boomgaarden H. News, Political Knowledge and Participation: The Differential Effects of News Media Exposure on Political Knowledge and Participation. Acta Polit. 2006;41(4):317–41. doi: 10.1057/palgrave.ap.5500164

[37] Granderath JS, Sondermann C, Martin A, Merkt M. Actual and Perceived Knowledge About COVID-19: The Role of Information Behavior in Media. Front Psychol. 2021;12:778886. doi: 10.3389/fpsyg.2021.778886 34975667 PMC8714891

[38] Martin JD, Sharma K. What Do Newsjunkies Consume and What Do They Know? Two Studies on Intrinsic Need For Orientation, News Diets, and Political Knowledge. International Journal of Public Opinion Research. 2023;35(1). doi: 10.1093/ijpor/edad002

[39] Ceci SJ. How much does schooling influence general intelligence and its cognitive components? A reassessment of the evidence. Developmental Psychology. 1991;27(5):703–22. doi: 10.1037/0012-1649.27.5.703

[40] Kahan DM, Peters E, Wittlin M, Slovic P, Ouellette LL, Braman D, et al. The polarizing impact of science literacy and numeracy on perceived climate change risks. Nature Clim Change. 2012;2(10):732–5. doi: 10.1038/nclimate1547

[41] Stroud NJ. Media use and political predispositions: revisiting the concept of selective exposure. Polit Behav. 2008;30:341–66. doi: 10.1007/s11109-007-9050-9

[42] Scott R. Does university make you more liberal? Estimating the within-individual effects of higher education on political values. Elect Stud. 2022;77:102471. doi: 10.1016/j.electstud.2022.102471

[43] Bilendi & respondi marktforschungsanbieter.de - Profiles. marktforschungsanbieter.de. https://marktforschungsanbieter.de/profiles/view/319 2023. Accessed 2023 June 27.

[44] Moshagen M, Bader M. semPower: General power analysis for structural equation models. Behav Res Methods. 2024;56(4):2901–22. doi: 10.3758/s13428-023-02254-7 37950114 PMC11522193

[45] Zimny L, Schroeders U, Wilhelm O. Ant colony optimization for parallel test assembly. Behav Res Methods. 2024;56(6):5834–48. doi: 10.3758/s13428-023-02319-7 38277085 PMC11335849

[46] Goecke B, Staab M, Schittenhelm C, Wilhelm O. Stop Worrying about Multiple-Choice: Fact Knowledge Does Not Change with Response Format. J Intell. 2022;10(4):102. doi: 10.3390/jintelligence10040102 36412782 PMC9680349

[47] Rodriguez MC. Three Options Are Optimal for Multiple‐Choice Items: A Meta‐Analysis of 80 Years of Research. Educational Measurement. 2005;24(2):3–13. doi: 10.1111/j.1745-3992.2005.00006.x

[48] Guo H, Zu J, Kyllonen P. A Simulation‐Based Method for Finding the Optimal Number of Options for Multiple‐Choice Items on a Test. ETS Research Report Series. 2018;2018(1):1–17. doi: 10.1002/ets2.12209

[49] Kline P. A handbook of test construction: Introduction to psychometric design. 1986.

[50] Lord FM. The Relation of the Reliability of Multiple-Choice Tests to the Distribution of Item Difficulties. Psychometrika. 1952;17(2):181–94. doi: 10.1007/bf02288781

[51] Olaru G, Witthöft M, Wilhelm O. Methods matter: Testing competing models for designing short-scale Big-Five assessments. J Res Personal. 2015;59:56–68. doi: 10.1016/j.jrp.2015.09.001

[52] Little TD, Slegers DW, Card NA. A non-arbitrary method of identifying and scaling latent variables in SEM and MACS models. Structural Equation Modeling: A Multidisciplinary Journal. 2006;13:59–72. doi: 10.1207/s15328007sem1301_3

[53] McDonald RP. Test theory: A unified treatment. Mahwah: Erlbaum. 1990.

[54] Bentler PM. Comparative fit indices in structural models. Psychol Bull. 1990;107:238–46. 10.1037/0033-2909.107.2.238 2320703

[55] Hu L, Bentler PM. Cutoff criteria for fit indexes in covariance structure analysis: Conventional criteria versus new alternatives. Struct Equ Model Multidiscip J. 1999;6:1–55. doi: 10.1080/10705519909540118

[56] Steiger JH. Structural model evaluation and modification: An interval estimation approach. Multivar Behav Res. 1990;25:173–80. 10.1207/s15327906mbr2502_4 26794479

[57] McNeish D, Wolf MG. Dynamic fit index cutoffs for confirmatory factor analysis models. Psychol Methods. 2023;28(1):61–88. doi: 10.1037/met0000425 34694832

[58] McNeish D, Wolf MG. Direct discrepancy dynamic fit index cutoffs for arbitrary covariance structure models. Structural Equation Modeling: A Multidisciplinary Journal. 2024;31:835–62. doi: 10.1080/10705511.2024.2308005

[59] R Core Team. R: A language and environment for statistical computing. Vienna, Austria: R Foundation for Statistical Computing. 2020.

[60] Revelle WR. Psych: Procedures for personality and psychological research. 2017.

[61] Rosseel Y. lavaan: An R package for structural equation modeling. J Stat Softw. 2012;48. doi: 10.18637/jss.v048.i02

[62] Wolf MG, McNeish D. dynamic : An R Package for Deriving Dynamic Fit Index Cutoffs for Factor Analysis. Multivariate Behav Res. 2023;58(1):189–94. doi: 10.1080/00273171.2022.2163476 36787513

[63] Ritchie SJ, Tucker-Drob EM. How Much Does Education Improve Intelligence? A Meta-Analysis. Psychol Sci. 2018;29(8):1358–69. doi: 10.1177/0956797618774253 29911926 PMC6088505

[64] Wilhelm O, Schroeders U. The Psychology of Human Thought. Heidelberg University Publishing. 2019. doi: 10.17885/HEIUP.470.C6677

[65] McGrew KS. CHC theory and the human cognitive abilities project: Standing on the shoulders of the giants of psychometric intelligence research. Intelligence. 2009;37(1):1–10. doi: 10.1016/j.intell.2008.08.004

[66] Cattell RB. The measurement of adult intelligence. Psychol Bull. 1943;40:153–93.

[67] Hambrick DZ, Engle RW. Effects of domain knowledge, working memory capacity, and age on cognitive performance: an investigation of the knowledge-is-power hypothesis. Cogn Psychol. 2002;44(4):339–87. doi: 10.1006/cogp.2001.0769 12018938

[68] Moehring A, Schroeders U, Wilhelm O. Knowledge Is Power for Medical Assistants: Crystallized and Fluid Intelligence As Predictors of Vocational Knowledge. Front Psychol. 2018;9:28. doi: 10.3389/fpsyg.2018.00028 29449819 PMC5799592

[69] Simonsmeier BA, Flaig M, Deiglmayr A, Schalk L, Schneider M. Domain-specific prior knowledge and learning: A meta-analysis. Educ Psychol. 2021. doi: 10.1080/00461520.2021.1939700

[70] Ackerman PL, Heggestad ED. Intelligence, personality, and interests: Evidence for overlapping traits. Psychol Bull. 1997;121(2):219–45. doi: 10.1037/0033-2909.121.2.219 9100487

[71] Lazowski RA, Hulleman CS. Motivation interventions in education: A meta-analytic review. Rev Educ Res. 2016;86:602–40. doi: 10.3102/0034654315617832

[72] Schneider M, Simonsmeier BA. How does prior knowledge affect learning? A review of 16 mechanisms and a framework for future research. Learn Individ Differ. 2025;122:102744. doi: 10.1016/j.lindif.2025.102744

[73] OECD. PISA 2018 results (volume VI): are students ready to thrive in an interconnected world?. Paris: OECD. 2020. doi: 10.1787/d5f68679-en

[74] Rosling H, Rosling O, Rönnlund AR. Factfulness: ten reasons we’re wrong about the world - and why things are better than you think. Paperback ed. London: SCEPTRE. 2019.

[75] Todd PM, Gigerenzer G. Précis of Simple heuristics that make us smart. Behav Brain Sci. 2000;23(5):727–41; discussion 742-80. doi: 10.1017/s0140525x00003447 11301545

[76] Schroeders U, Wilhelm O, Olaru G. The influence of item sampling on sex differences in knowledge tests. Intelligence. 2016;58:22–32. doi: 10.1016/j.intell.2016.06.003

[77] Reysen S, Katzarska-Miller I. The psychology of global citizenship: A review of theory and research. Lanham, MD, US: Lexington Books/Rowman & Littlefield. 2018.

[78] Ellen PS. Do we know what we need to know? Objective and subjective knowledge effects on pro-ecological behaviors. J Bus Res. 1994;30:43–52. doi: 10.1016/0148-2963(94)90067-1

[79] Olderbak S, Wilhelm O. Overarching principles for the organization of socioemotional constructs. Curr Dir Psychol Sci. 2020;29:63–70. doi: 10.1177/0963721419884317

[80] Pfattheicher S, Geiger M, Hartung J, Weiss S, Schindler S. Old wine in new bottles? The case of self–compassion and neuroticism. Eur J Personal. 2017;31:160–9. doi: 10.1002/per.2097

[81] Bethlehem J. Selection bias in web surveys. Int Stat Rev. 2010;78:161–88. doi: 10.1111/j.1751-5823.2010.00112.x

[82] Callegaro M, Villar A, Yeager D, Krosnick JA. A critical review of studies investigating the quality of data obtained with online panels based on probability and nonprobability samples1. Online Panel Research. Wiley. 2014. 23–53. doi: 10.1002/9781118763520.ch2

[83] Dawum. Bundestagswahl: Wahlumfrage vom 20.01.2024 von INSA. dawum.de. https://dawum.de/Bundestag/INSA/2024-01-20/ 2024. Accessed 2026 May 27.

[84] Ehni E. ARD-DeutschlandTrend: AfD erreicht neuen Bestwert. tagesschau.de. https://www.tagesschau.de/inland/deutschlandtrend/deutschlandtrend-3372.html 2023. Accessed 2026 May 27.

[85] Uziel L. Rethinking Social Desirability Scales: From Impression Management to Interpersonally Oriented Self-Control. Perspect Psychol Sci. 2010;5(3):243–62. doi: 10.1177/1745691610369465 26162157

