# Supplementary material for: Measuring planetary health knowledge: Development and psychometric evaluation of a taxonomy-based test
Source: PLoS One. 2026 Oct 6;21(10):e0359156. doi: 10.1371/journal.pone.0359156 (PMC13641387; doi:10.1371/journal.pone.0359156)
Supplement: Supplementary file 1 [file pone.0359156.s001.docx]

| S1 Table. Number of items in initial and final item sets in seven PHK domains | | | | | | | |
| --- | --- | --- | --- | --- | --- | --- | --- |
|  |  |  | All items | p-items | g-items | f-items | c-items |
| P1 | Initial itemset | All items | 60 | 14 | 13 | 18 | 15 |
|  |  | Subdomain A | 18 | 2 | 5 | 6 | 5 |
|  |  | Subdomain B | 29 | 10 | 6 | 6 | 7 |
|  |  | Subdomain C | 13 | 2 | 2 | 6 | 3 |
|  | Itemset after item reduction | All items | 25 | 2 | 3 | 9 | 11 |
|  |  | Subdomain A | 10 | 0 | 1 | 5 | 4 |
|  |  | Subdomain B | 11 | 1 | 2 | 3 | 5 |
|  |  | Subdomain C | 4 | 1 | 0 | 1 | 2 |
| P2 | Initial itemset | All items | 60 | 18 | 15 | 12 | 15 |
|  |  | Subdomain A | 19 | 2 | 5 | 6 | 5 |
|  |  | Subdomain B | 27 | 10 | 6 | 6 | 7 |
|  |  | Subdomain C | 14 | 2 | 2 | 6 | 3 |
|  | Itemset after item reduction | All items | 25 | 0 | 8 | 7 | 7 |
|  |  | Subdomain A | 6 | 0 | 0 | 3 | 3 |
|  |  | Subdomain B | 11 | 0 | 5 | 2 | 2 |
|  |  | Subdomain C | 8 | 0 | 3 | 2 | 2 |

| S1 Table *(continued)* | | | | | | | |
| --- | --- | --- | --- | --- | --- | --- | --- |
|  |  |  | All items | p-items | g-items | f-items | c-items |
| P3 | Initial itemset | All items | 60 | 17 | 15 | 18 | 10 |
|  |  | Subdomain A | 13 | 5 | 3 | 2 | 3 |
|  |  | Subdomain B | 37 | 10 | 10 | 11 | 6 |
|  |  | Subdomain C | 10 | 2 | 2 | 5 | 1 |
|  | Itemset after item reduction | All items | 30 | 6 | 8 | 9 | 7 |
|  |  | Subdomain A | 10 | 3 | 3 | 2 | 2 |
|  |  | Subdomain B | 14 | 2 | 4 | 4 | 4 |
|  |  | Subdomain C | 6 | 1 | 1 | 3 | 1 |
| P4 | Initial itemset | All items | 82 | 19 | 20 | 19 | 24 |
|  |  | Subdomain A | 21 | 5 | 6 | 4 | 6 |
|  |  | Subdomain B | 19 | 6 | 4 | 3 | 6 |
|  |  | Subdomain C | 22 | 4 | 6 | 6 | 6 |
|  |  | Subdomain D | 20 | 4 | 4 | 6 | 6 |
|  | Itemset after item reduction | All items | 37 | 4 | 7 | 11 | 15 |
|  |  | Subdomain A | 9 | 0 | 3 | 3 | 3 |
|  |  | Subdomain B | 7 | 0 | 2 | 1 | 4 |
|  |  | Subdomain C | 11 | 2 | 1 | 4 | 5 |
|  |  | Subdomain D | 10 | 2 | 1 | 4 | 3 |

| S1 Table *(continued)* | | | | | | | |
| --- | --- | --- | --- | --- | --- | --- | --- |
|  |  |  | All items | p-items | g-items | f-items | c-items |
| P5 | Initial itemset | All items | 81 | 19 | 20 | 20 | 22 |
|  |  | Subdomain A | 33 | 7 | 8 | 8 | 10 |
|  |  | Subdomain B | 22 | 5 | 4 | 6 | 7 |
|  |  | Subdomain C | 26 | 7 | 8 | 6 | 5 |
|  | Itemset after item reduction | All items | 36 | 4 | 7 | 10 | 15 |
|  |  | Subdomain A | 17 | 2 | 4 | 3 | 8 |
|  |  | Subdomain B | 11 | 1 | 2 | 4 | 4 |
|  |  | Subdomain C | 8 | 1 | 1 | 3 | 3 |
| P6 | Initial itemset | All items | 81 | 21 | 24 | 18 | 18 |
|  |  | Subdomain A | 33 | 8 | 10 | 7 | 8 |
|  |  | Subdomain B | 23 | 7 | 6 | 6 | 4 |
|  |  | Subdomain C | 25 | 6 | 8 | 5 | 6 |
|  | Itemset after item reduction | All items | 36 | 3 | 9 | 10 | 14 |
|  |  | Subdomain A | 17 | 3 | 4 | 4 | 6 |
|  |  | Subdomain B | 11 | 0 | 3 | 4 | 4 |
|  |  | Subdomain C | 8 | 0 | 2 | 2 | 4 |

| S1 Table *(continued)* | | | | | | | |
| --- | --- | --- | --- | --- | --- | --- | --- |
|  |  |  | All items | p-items | g-items | f-items | c-items |
| P7 | Initial itemset | All items | 79 | 18 | 21 | 21 | 19 |
|  |  | Subdomain A | 20 | 6 | 7 | 5 | 2 |
|  |  | Subdomain B | 20 | 4 | 4 | 7 | 5 |
|  |  | Subdomain C | 19 | 6 | 4 | 4 | 5 |
|  |  | Subdomain D | 20 | 2 | 6 | 5 | 7 |
|  | Itemset after item reduction | All items | 47 | 7 | 8 | 16 | 16 |
|  |  | Subdomain A | 8 | 2 | 0 | 4 | 2 |
|  |  | Subdomain B | 15 | 2 | 3 | 7 | 3 |
|  |  | Subdomain C | 12 | 2 | 3 | 2 | 5 |
|  |  | Subdomain D | 12 | 1 | 2 | 3 | 4 |
| *Note.* p-items = prevalence, g-items = global distribution, f-items = fact knowledge, c-items = comprehension. P1 Subdomains: A = Health Management, B = Physical Health, C = Mental Health. P2 Subdomains: A = Food & Water Management, B = Sustainable Food Production, C = Malnutrition. P3 Subdomains: A = Nature & Nature Conservation, B = Environmental Change, C = Climate Action. P4 Subdomains: A = Peace, B = Everyday Security, C = Human Rights Violations, D = Natural Disasters. P5 Subdomains: A = Basic Education, B = Secondary Education, C = Academic & Vocational Education. P6 Subdomains: A = Wealth, B = Possessions, C = Mobility. P7 Subdomains: A = Participation, B = Rule of Law, C = Welfare, D = Economy & Industry. | | | | | | | |
