# Supplementary material for: Measuring planetary health knowledge: Development and psychometric evaluation of a taxonomy-based test
Source: PLoS One. 2026 Oct 6;21(10):e0359156. doi: 10.1371/journal.pone.0359156 (PMC13641387; doi:10.1371/journal.pone.0359156)
Supplement: Supplementary file 2 [file pone.0359156.s002.docx]

| **S2 Table.** **Model fit indices for covariate models in studies 1 to 7.** | | | | | | | | | | | | | | | | |
| --- | --- | --- | --- | --- | --- | --- | --- | --- | --- | --- | --- | --- | --- | --- | --- | --- |
|  |  |  | |  | |  |  | |  | |  | |  |  | *3DFI cutoff (mediocre fit)* | |
|  |  | *Chi2* | *df* | *p* | *CFI* | | | *TLI* | | *RMSEA* | | *SRMR* | |  | *CFI* | *RMSEA* |
| P1 | gc | 59 | 54 | .286 | .991 | | | .988 | | .018 | | .070 | |  | .970 | .034 |
|  | GCI | 57 | 19 | .000 | .957 | | | .937 | | .081 | | .046 | |  | .958 | .095 |
|  | Behavior | 31 | 16 | .014 | .956 | | | .924 | | .056 | | .050 | |  | .958 | .058 |
| P2 | gc | 71 | 54 | .062 | .975 | | | .969 | | .032 | | .076 | |  | .975 | .032 |
|  | GCI | 78 | 19 | .000 | .927 | | | .892 | | .101 | | .053 | |  | .932 | .115 |
|  | Behavior | 33 | 16 | .007 | .927 | | | .872 | | .060 | | .052 | |  | .931 | .063 |
| P3 | gc | 85 | 54 | .004 | 1.000 | | | 1.027 | | .000 | | .057 | |  | .975 | .032 |
|  | GCI | 50 | 19 | .000 | .953 | | | .931 | | .072 | | .049 | |  | .976 | .063 |
|  | Behavior | 77 | 16 | .000 | .918 | | | .858 | | .074 | | .057 | |  | .973 | .080 |
| P4 | gc | 59 | 54 | .310 | .991 | | | .989 | | .017 | | .072 | |  | .991 | .017 |
|  | GCI | 53 | 19 | .000 | .966 | | | .950 | | .077 | | .040 | |  | .968 | .086 |
|  | Behavior | 279 | 79 | .000 | .871 | | | .845 | | .065 | | .078 | |  | .849 | .078 |
| P5 | gc | 58 | 54 | .345 | .993 | | | .992 | | .015 | | .072 | |  | .993 | .015 |
|  | GCI | 44 | 19 | .001 | .959 | | | .939 | | .067 | | .045 | |  | .974 | .075 |
|  | Behavior | 279 | 79 | .000 | .886 | | | .863 | | .063 | | .074 | |  | .893 | .076 |
| P6 | gc | 55 | 54 | .449 | .999 | | | .999 | | .006 | | .066 | |  | .979 | .006 |
|  | GCI | 49 | 19 | .000 | .955 | | | .933 | | .072 | | .049 | |  | .957 | .084 |
|  | Behavior | 279 | 79 | .000 | .929 | | | .914 | | .045 | | .058 | |  | .838 | .079 |
| P7 | gc | 66 | 54 | .132 | .979 | | | .975 | | .028 | | .084 | |  | .970 | .028 |
|  | GCI | 38 | 19 | .006 | .968 | | | .953 | | .060 | | .039 | |  | .984 | .073 |
|  | Behavior | 279 | 79 | .000 | .872 | | | .846 | | .061 | | .074 | |  | .859 | .061 |
| *Note.* Due to differences in the assessed items, behavior was modeled as a bifactor model in domains P1, P2, and P3 and as three correlated factors in domains P4, P5, P6, and P7. Values in green indicate that empiric indices exceed the 3DFI cutoffs for mediocre fit, values in red indicate empiric values are worse than 3DFI cutoffs for mediocre fit. | | | | | | | | | | | | | | | | |
