# Supplementary material for: Measuring planetary health knowledge: Development and psychometric evaluation of a taxonomy-based test
Source: PLoS One. 2026 Oct 6;21(10):e0359156. doi: 10.1371/journal.pone.0359156 (PMC13641387; doi:10.1371/journal.pone.0359156)
Supplement: Supplementary file 3 [file pone.0359156.s003.docx]

| S3 Table. Manifest PHK scores for individual and grouped party groups in studies 1 to 7. | | | | | | | | | | | | | | | | | | | | | | | | | | | | |
| --- | --- | --- | --- | --- | --- | --- | --- | --- | --- | --- | --- | --- | --- | --- | --- | --- | --- | --- | --- | --- | --- | --- | --- | --- | --- | --- | --- | --- |
|  |  | **P1: Health** | | |  | **P2: Nutrition** | | |  | **P3: Environment** | | |  | **P4: Safety** | | |  | **P5: Education** | | |  | **P6: Standard of Living** | | |  | **P7: Political Systems** | | |
| **Party** |  | ***N*** | ***Mean*** | ***Sd*** |  | ***N*** | ***Mean*** | ***Sd*** |  | ***N*** | ***Mean*** | ***Sd*** |  | ***N*** | ***Mean*** | ***Sd*** |  | ***N*** | ***Mean*** | ***Sd*** |  | ***N*** | ***Mean*** | ***Sd*** |  | ***N*** | ***Mean*** | ***Sd*** |
| Left* |  | 15 | .533 | .157 |  | 15 | .661 | .118 |  | 18 | .556 | .206 |  | 20 | .650 | .160 |  | 14 | .692 | .094 |  | 13 | .556 | .139 |  | 11 | .735 | .130 |
| Greens |  | 52 | .663 | .180 |  | 35 | .683 | .182 |  | 32 | .690 | .151 |  | 35 | .677 | .153 |  | 25 | .629 | .189 |  | 31 | .694 | .132 |  | 30 | .700 | .156 |
| SPD |  | 49 | .597 | .149 |  | 48 | .542 | .195 |  | 55 | .533 | .192 |  | 45 | .568 | .173 |  | 46 | .586 | .199 |  | 42 | .586 | .170 |  | 40 | .661 | .179 |
| FDP* |  | 16 | .560 | .195 |  | 14 | .557 | .250 |  | 9 | .522 | .159 |  | 9 | .661 | .131 |  | 10 | .567 | .237 |  | 14 | .573 | .101 |  | 15 | .558 | .195 |
| CDU/ CSU* |  | 49 | .558 | .159 |  | 59 | .571 | .177 |  | 58 | .556 | .171 |  | 56 | .615 | .181 |  | 76 | .569 | .174 |  | 70 | .598 | .169 |  | 68 | .602 | .194 |
| AfD |  | 64 | .551 | .172 |  | 64 | .583 | .182 |  | 73 | .496 | .179 |  | 68 | .566 | .172 |  | 55 | .503 | .177 |  | 69 | .584 | .174 |  | 54 | .522 | .193 |
| Others |  | 58 | .588 | .142 |  | 69 | .552 | .182 |  | 63 | .522 | .185 |  | 66 | .601 | .160 |  | 70 | .541 | .166 |  | 61 | .629 | .148 |  | 57 | .501 | .174 |
| CDU/CSU/FDP |  | 65 | .559 | .167 |  | 73 | .568 | .191 |  | 67 | .551 | .169 |  | 65 | .622 | .175 |  | 86 | .569 | .181 |  | 84 | .594 | .159 |  | 83 | .594 | .193 |
| Others/ Left* |  | 73 | .576 | .145 |  | 84 | .572 | .177 |  | 81 | .530 | .189 |  | 86 | .612 | .161 |  | 84 | .566 | .166 |  | 74 | .616 | .148 |  | 68 | .539 | .188 |
| *Note.* Others implied non-voters. Party groups marked with * were not reported in the main analyses of the manuscript as voters of the Left were grouped with Others/non-voters, whereas voters of the FDP were grouped with CDU/CSU. | | | | | | | | | | | | | | | | | | | | | | | | | | | | |
